# Considerations and Caveats in Combating ESKAPE Pathogens against Nosocomial Infections

**DOI:** 10.1002/advs.201901872

**Published:** 2019-12-05

**Authors:** Yu‐Xuan Ma, Chen‐Yu Wang, Yuan‐Yuan Li, Jing Li, Qian‐Qian Wan, Ji‐Hua Chen, Franklin R. Tay, Li‐Na Niu

**Affiliations:** ^1^ State Key Laboratory of Military Stomatology National Clinical Research Center for Oral Diseases Shaanxi Key Laboratory of Stomatology Department of Prosthodontics School of Stomatology The Fourth Military Medical University 145 Changle West Road Xi'an Shaanxi 710032 P. R. China; ^2^ The Graduate School Augusta University 1430, John Wesley Gilbert Drive Augusta GA 30912‐1129 USA

**Keywords:** antimicrobial peptides, antiresistance, antivirulence, bacteriophages, nanodelivery strategies

## Abstract

ESKAPE pathogens (*Enterococcus faecium*, *Staphylococcus aureus*, *Klebsiella pneumoniae*, *Acinetobacter baumannii*, *Pseudomonas aeruginosa*, and *Enterobacter* species) are among the most common opportunistic pathogens in nosocomial infections. ESKAPE pathogens distinguish themselves from normal ones by developing a high level of antibiotic resistance that involves multiple mechanisms. Contemporary therapeutic strategies which are potential options in combating ESKAPE bacteria need further investigation. Herein, a broad overview of the antimicrobial research on ESKAPE pathogens over the past five years is provided with prospective clinical applications.

## Graphical abstract

ESKAPE pathogens (*Enterococcus faecium*, *Staphylococcus aureus*, *Klebsiella pneumoniae*, *Acinetobacter baumannii*, *Pseudomonas aeruginosa*, and *Enterobacter* species) are among the most common opportunistic pathogens in nosocomial infections. Herein, contemporary therapeutic strategies against drug‐resistance ESKAPE and antimicrobial agents with prospective clinical applications are summarized, with a forecast on the future tendency of ESKAPE infection control.

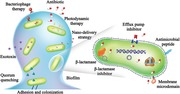

## 1 Introduction

Injudicious use of antibiotics has created unprecedented challenges for the human civilization because of escalation of the antimicrobial resistance. Antimicrobial resistance is a natural phenomenon when microbes are exposed to antimicrobial drugs. Not only the overuse of antibiotics in health care, agriculture, and the environment^1^ but also the inappropriate antibiotic consumption, such as inappropriate choices, inadequate dosing, poor adherence to treatment guidelines, contribute to the increasing antimicrobial resistance selection.^2^ What's more, the antibiotic treatment for hard‐to‐treat multidrug‐resistant bacterial infections is limited.^1^ The main reasons include the incomprehensive consideration of resistance mechanisms,^3^ a lack of new drug development due to reduced economic incentives, and challenge from regulatory requirements. ESKAPE pathogens (*Enterococcus faecium*, *Staphylococcus aureus*, *Klebsiella pneumoniae*, *Acinetobacter baumannii*, *Pseudomonas aeruginosa*, and *Enterobacter* species) are among the most common opportunistic pathogens in nosocomial infections.^4^ The acronym ESKAPE reflects the ability of these organisms to “escape” killing by antibiotics and defy eradication by conventional therapies, which accounts for extensive morbidity and mortality for patients and increased resource utilization in healthcare.^5^ Infections associated with ESKAPE have become a major problem in the choice of effective therapeutic strategies.

**Figure 1:**
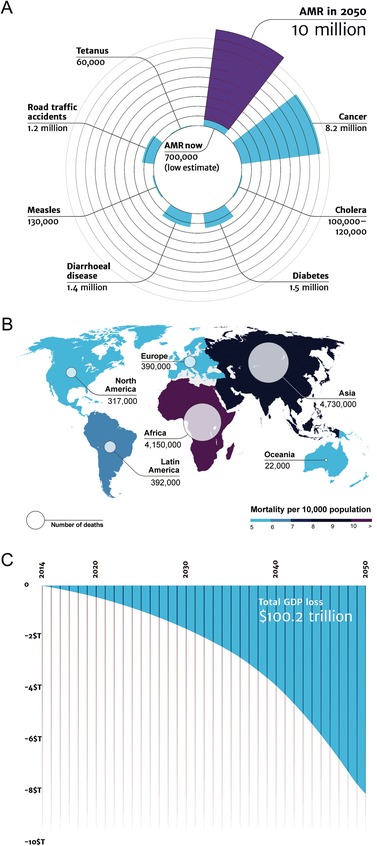
Impact of antimicrobial resistance. Two teams from Europe modeled the increase in the rates of antimicrobial resistance (AMR) based on the information available in 2014, each using their own methodology, to understand the impact of AMR would have on the world population and its economic output. A) Deaths attributable to AMR every year compared to other major causes of death. The estimated number of AMR will increase to 10 million by 2050, approaching the total number of deaths caused by all diseases today. B) Deaths attributable to AMR in different parts of the world by 2050. There is a tendency for reduced mortality continents with better economic conditions and more stringent antibiotic management. C) The impact of AMR on the world's economy between 2014 and 2050 (in trillions of US dollars) predicts an exponentially loss in gross domestic product (GDP) attributable to combating AMR. Reproduced under the terms of the Creative Commons Attribution 4.0 International Public License.^8^ Copyright 2016, Review on Antimicrobial Resistance.

ESKAPE pathogens are associated with a high risk of mortality and increased economic costs.^6^ The U.S. Centers for Disease Control and Prevention (CDC) estimated that antibiotic‐resistant microorganisms cause more than two million infections in the United States each year, resulting in at least 23 000 deaths.^7^ Globally, the number of antimicrobial resistance per year is expected to increase ten times by 2050 (**Figure** **1**A) with the projected scenario of deaths varying among different continents (Figure 1B).^8^ The total estimated cost in fighting resistance to five pathogens (*S. aureus, E. coli, K. pneumoniae, A. baumanii, and P. aeruginosa*) was $0.5 billion and $2.9 billion in Thailand and the United States, respectively.^9^ Notable, global antibiotic consumption increased by 65% from 2000 to 2015, which was primarily driven by low/middle income countries.^10^ A reduction of 2–3.5% in gross domestic product caused by investments on dealing with such infections has been forecasted, reaching 100 trillion US dollars by 2050 (Figure 1C).^8^

The golden era for medicine to treat bacterial infectious diseases has elapsed. Drug‐resistant bacteria have threatened to eradicate antibiotics from an already shrinking repertoire of therapeutic arsenal.^11^ Colistin has been regarded as one of the last resort antibiotics to treat severe infections caused by the carbapenem‐resistant *Enterobacteriaceae* because of the previously perceived low rate of chromosomally mediated drug resistance in the family of bacteria.^12^ Since the report of the polymixin‐resistant gene MCR‐1 in *Enterobacteriaceae* isolates from animals and humans that is responsible for plasmid‐mediated colistin resistance,^13^ there has been numerous reports identifying the rapid distribution of this transmissible resistance mechanism.^14^

Despite universal agreement that antibiotic overprescribing is a problem, the practice continues vexing. For example, of the 40 million antibiotic visits prescribed for respiratory conditions in ambulatory care in the United States between 2007 and 2009, there were 27 million visits (67.5%) in which antibiotics were prescribed unnecessarily.^15^ One would have thought that the issue of antibiotic misuse would have declined substantially with intense promotions from national and international health organizations. However, a study published in 2019 reported that among all outpatient antibiotic prescriptions of 19+ million privately insured USA children and nonelderly adults in 2016, 23.2% were considered inappropriate, 35.5% were potentially appropriate, and 28.5% were not associated with a recent diagnosis code (total 87.2%).^16^

Apart from the loose way in which antibiotics are prescribed, antibiotic resistance may also be caused by survival competition within the bacteria consortia. Bacteria have developed two types of resistance to evade the action of antibiotics. “Intrinsic or innate resistance” refers to a natural insensitivity in certain bacterial strains that have never been susceptible to a particular antibiotic. For example, *P. aeruginosa* is intrinsically resistant to many classes of antibiotics because there are few of porins in its outer membrane, thus many antibiotics cannot penetrate the interior of these bacteria cells. In contrast, “acquired resistance” represents a more devastating circumstance in which bacteria become resistant to a particular antibiotic to which it was previously susceptible. This can result from mutation or exchange of genetic materials between bacteria. For example, nosocomial outbreaks have been reported across the globe that are attributed to the development of increasingly resistant strains of *A. baumannii* and *P. aeruginosa*.^17^ These bacteria are intrinsically resistant to many antibiotics due to their natural membrane impermeability, basal efflux activity, and the production of inducible β‐lactamases. In addition, they can develop acquired resistance via mutations of preexisting genes and horizontal transfer of resistance determinants.^18^

Development of novel anti‐infective strategies against ESKAPE pathogens is consistently being pursued.^19^ Multidrug‐resistant (MDR), extensively drug‐resistant (XDR), and pan drug‐resistant (PDR) bacteria have been well defined according to the standardized international terminology developed by European Center for Disease Control (ECDC) and the CDC in USA.^20^ Multidrug resistance is defined as acquired nonsusceptibility to at least one agent in three or more antimicrobial categories. Extensively drug resistance is defined as nonsusceptibility to at least one agent in all but two or fewer antimicrobial categories (i.e., bacterial isolates remain susceptible to only one or two antimicrobial categories). Pan drug resistance is defined as nonsusceptibility to all agents in all antimicrobial categories. In the present review, therapeutic strategies that are potentially effective in combating ESKAPE bacteria in the postantibiotic era are presented.

Discussion topics include new drugs in the existing classes of antibiotics; antiresistance drugs which are administered as antibiotic adjuvants to potentiate the effects of current antimicrobials; antivirulence drugs which are directed against bacterial virulence factors; as well as alternative treatments, which include strategies such as antimicrobial peptides (AMPs), nanodelivery strategies, bacteriophage therapy, photodynamic therapy, and other novel antimicrobial drugs. The order of topics was basically based on the quality of articles published in the last five years and corresponding clinical application prospects.

## 2 Incidence of MDR, XDR, and PDR in ESKAPE Pathogens

Infections caused by the ESKAPE result in high mortality and morbidity rates, high healthcare costs, diagnostic dilemma, and difficulty in the initiation of empirical treatment.^21^ A request was made to the World Health Organization by member states in 2017 to develop a global priority pathogen list (PPL) of antibiotic‐resistant bacteria to help prioritize research and develop of new and effective antibiotic treatments.^22^ Because of the high prevalence of multidrug resistance among ESKAPE pathogens, they are prominently featured in the global PPL of antibiotic‐resistant bacteria. The global PPL stratifies bacterial pathogens into three priority tiers: critical, high, and medium. Carbapenem‐resistant *A. baumannii*, *P. aeruginosa*, and *Enterobacteriaceae* species, which include *K. pneumonia*, are listed in the critical priority tier. Methicillin‐resistant and vancomycin‐intermediate/resistant *S. aureus*, in addition to *E. faecium*, are listed in the high priority tier. **Table** **1** shows the incidence of MDR, XDR, and PDR ESKAPE pathogens reported in recent epidemiology and prevalence studies.

**Table 1:** Proportion of clinical ESKAPE isolates and their proportion segregating in MDR, XDR, and PDR as defined by the CDC/ECDC panel

Country	Collection period	Study population	*E. faecalis*	*S. aureus*	*K. pneumoniae*	*A. baumannii*	*P. aeruginosa*	*Enterobacter* spp.	*E. coli*	Refs.
India	January to December, 2015	5103 resistance records from 4437 patients	5.9%	5.5%	26.9%	10.2%	11.6%	2.6%	37.4%	23
			MDR/XDR	MDR/XDR	MDR/XDR	MDR/XDR	MDR/XDR	MDR/XDR	MDR/XDR	
India	April 15 to July 15, 2014	1060 bacterial strains from 9304 patients	4.2%	23.8%	18.9%	4.0%	20.0%	2.9%	24.6%	24
			28.9% MDR 35.6% XDR.	49.6% MDR 15.1% XDR	36% MDR 11% XDR	45.2% MDR 19% XDR	37.5% MDR 12.5% XDR	35.5% MDR 19.4% XDR	30.3% MDR 8.4% XDR	
Kuwait	January to December, 2017	201 patients with burn injury	4.9%	14.6%	19.5%	41.5%	14.6%	–	–	25
			0% MDR	66.7% MDR	50.0% MDR	100.0% MDR	16.7% MDR	–	–	
China	January 2012 to December 2014	7579 patients with hospital‐acquired infections	2.1%	14.9%	14.5%	15.3%	15.4%	–	29.0%	26
			1.3% MDR	1.3% MDR	41.9% MDR	50.7% MDR	37.6% MDR	–	55.4% MDR	
Indonesia	January 2015 to December 2016	299 positive blood samples from 2542 pediatric patients	2.7%	3.0%	–	8.0%	1.7%	18.1%	–	27
			75% MDR 25% XDR	88.9% MDR 11.1% XDR	–	83.3% MDR 16.7% XDR	100% MDR	87.0% MDR 13.0% XDR	–	
South Africa	August 2011 to December 2015	64502 ESKAPE clinical isolates	3.4%	38.0%	22.2%	12.4%	17.4%	6.6%	–	28
			–	24.6% MRSA	–	79.2% were MDR	–	–	–	
Nepal	November 2014 to August 2015	182 pus and fine needle aspirates collected from patients with clinical features of wound infection	*Enterococcus* spp. 4.3%	56.9%	5.2%	*Acinetobacter* spp. 5.2%	4.3%	–	8.6%	29
			80% MDR	80% MDR	80% MDR	66.7% MDR	66.7% MDR	–	80% MDR	
India	January 2012 to December 2016	993 identified pathogens from 2984 patients with healthcare associated infections	Enterococcus spp. 2.0%	*Staphylococcus* spp. *6.6%*	*Klebsiella* spp. 15.1%	*Acinetobacter* spp. 42.9%	*Pseudomonas* spp. 10.2%	–	11.7%	30
			–	–	–	88.0% MDR 61.9% XDR	88.0% MDR 61.9% XDR	–	–	
Spain	–	203 microbiological confirmations (from 343 patients)	for *S. aureus*, *Enterococcus* spp., *Enterobacteriaceae* (other than Salmonella and Shigella), *P. aeruginosa*, and *Acinetobacter* spp.							31
			44% MDR, 12% XDR, 3% PDR							
Saudi Arabia	2014–2015	155 patients positive for *E. faecalis* infection	100.0%	–	–	–	–	–	–	32
			96.1% MDR	–	–	–	–	–	–	
Ethiopia	May to September, 2016	126 bacterial etiologies isolated from 118 patients with healthcare associated infections	–	20.6%	*Klebsiella* species, 23.8%	1.6%	7.1%	4.8%	24.6%	33
			–	38.5% MDR 38.5% XDR 11.5% PDR	*Klebsiella* species; 30% MDR 43.3% XDR 6.7% PDR	50% XDR 50% PDR	22.2% MDR 44.4% XDR 33% PDR	33.3% MDR 50% XDR 16.7% PDR	35.5% MDR 32.3% XDR 22.6% PDR	
Romania	2010– 2012	1001 bacterial strains (of 1534 samples) from 2404 adult patients	–	21.8%	18.8%	14.1%	14.2%	18.4%	11.3%	34
			–	66.5% MDR 20.2% XDR	87.8% MDR 35.6% XDR	99.3% MDR 41.1% XDR	69.0% MDR 20.4% XDR	67.9% MDR 13.0% XDR	67.9% MDR 13.0% XDR	
Nigeria	June to September, 2015	201 mid‐stream urine samples from asymptomatic pregnant women	–	10.0%	22.4%	–	17.9%	–	9.0%; 100% MDR	35
			–	90% MDR	90% MDR	–	88.9% MDR	–		
Ethiopia	September to December, 2016	242 swabs of health care workers	–	12.0%	–	–	–	–	–	36
			–	48.3% MRSA	–	–	–	–	–	
China	August to November, 2015	Swabs from 1834 pregnant women and their neonates	–	12.1%	–	–	–	–	–	37
			–	53.0% MDR	–	–	–	–	–	
China	August to November, 2015	Serial swabs collected from 1834 mothers and their newborn infants	–	7.3% in mothers 3.3% in infants	–	–	–	–	–	38
			–	66.7% MDR in mothers 38.3% in infants	–	–	–	–	–	
Tanzania	June to October, 2016	379 nasal swabs from health care workers	–	41.4%	–	–	–	–	–	39
			–	38.9% MDR	–	–	–	–	–	
Afghanistan	September 2016 to February 2017	105 clinical strains of *S. aureus* isolated from hospitalized patients	–	100.0%	–	–	–	–	–	40
			–	91.4% MDR	–	–	–	–	–	
Mexico	January 1 to June 30, 2018	22943 strains from 47 Mexican centers	–	–	*Klebsiella* spp.: 14.5%	*Acinetobacter* spp.: 3.8%	8.7%	5.8%	50.9%	41
			–	–	22.6% MDR	53.0% MDR, 43.2% possible XDR 8.8% XDR 38.8% possible PDR	8.8% MDR 8.3% possible XDR 0.2% XDR 4.4% possible PDR	11.9% MDR	19.4% MDR 8.1% possible XDR 0.04% possible PDR	
China	January 1, 2007 to March 31, 2017	88 MDR/XDR bacteria from urinary tract specimens in 1569 kidney transplant recipients	–	–	17.0%	10.2%	2.3%	*Enterobacter aerogenes* 2.3%, *Enterobacter cloacae* 1.1%	62.5%	42
			–	–	MDR/XDR	MDR/XDR	MDR/XDR	MDR/XDR	MDR/XDR	
Egypt	November 2015 to October 2016	195 positive culture specimens from 529 febrile neutropenic cancer patients	–	–	16.4%	6.2%	3.1%	*Enterobacter cloacae* 46.2%	17.4%	43
			–	–	75% MDR	75% MDR	33.3% MDR	79.4% MDR	79.4% MDR	
China	January 1 2016 to October 1 2017	19 in‐patients with ventriculitis caused by *A. baumannii* or *K. pneumonia*	–	–	26.3%	73.7%	–	–	–	44
			–	–	80% MDR 20% XDR	85.7% MDR 14.3% XDR	–	–	–	
Tunisia	2010–2017	770 patients with community‐acquired urinary tract infections caused by *Enterobacteriaceae*	–	–	14.4%	–	–	100.0%	72.7%	45
			–	–	17.2% MDR	–	–	47.9% MDR	76.1% MDR	
Ethiopia	January 1 to May 30, 2017	426 *Enterobacteriaceae* isolates	–	–	24.10%	–	–	–	53.50%	46
			–	–	82.5% MDR	–	–	–	65.3% MDR	
Iran	2012–2013	100 clinical isolates of *K. pneumoniae*	–	–	100.0%	–	–	–	–	47
			–	–	56% MDR	–	–	–	–	
Spain	January 2014 to December 2016	1725 adult patients colonized by *K. pneumoniae* in an intensive care unit (ICU)	–	–	100%	–	–	–	–	48
			–	–	17.9% MDR	–	–	–	–	
Brazil	January 2014 to May 2015	25 *K. pneumoniae* clinical isolates collected from patients and devices at ICUs	–	–	100.0%	–	–	–	–	49
			–	–	84.0% MDR	–	–	–	–	
India	March 2017 to February 2018	357 blood culture samples identified with *Acinetobacter* sp. during hospitalization	–	–	–	13.4%	–	–	–	50
			–	–	–	95.9% MDR 93.8% XDR	–	–	–	
Lithuania	January 2014 to December 2015	60 patients with ventilator‐associated pneumonia in ICU due to drug‐resistant *A. baumannii*	–	–	–	100.0%	–	–	–	51
			–	–	–	13.3% MDR 68.3% XDR 18.3% possible PDR	–	–	–	
Iran	October 2015 to October 2016	147 nonduplicate *A. baumannii* isolates from clinical specimens	–	–	–	100.0%	–	–	–	52
			–	–	–	2.7% MDR 97.3% XDR	–	–	–	
India	2011–2014	741 clinical *Acinetobacter* spp. isolates	–	–	–	*Acinetobacter* spp. 100%	–	–	–	53
			–	–	–	MDR isolates 89.4– 95.9%	–	–	–	
Iran	January to June, 2015	96 samples detected with *P. aeruginosa* from 120 wound burn samples	–	–	–	–	80.0%	–	–	54
			–	–	–	–	95.8% MDR 87.5% XDR	–	–	
Iran	2013	88 *P. aeruginosa* isolates from patients	–	–	–	–	100.0%	–	–	55
			–	–	–	–	54.5% MDR 33% XDR	–	–	
Venezuela	2009–2016	176 strains from patients diagnosed with clinical infections	–	–	–	–	100.0%	–	–	56
			–	–	–	–	MDR and XDR strains increased from 2009 (24.2 and 4.8%) to 2016 (53.1 and 18.8%)	–	–	
Global	1997–2016	52 022 clinically *P. aeruginosa* isolates from ≥200 medical centers	–	–	–	–	100.0%	–	–	57
			–	–	–	–	24.9% MDR, 24.9% XDR, and 0.1% PDR	–	–	
Malaysia	2015	53 clinical isolates of *P. aeruginosa*	–	–	–	–	100.0%	–	–	58
			–	–	–	–	7.5% MDR	–	–	
China	January 2013 to December 2016	157 patients with hospital‐acquired pneumonia caused by *P. aeruginosa*	–	–	–	–	100.0%	–	–	59
			–	–	–	–	43.9% MDR	–	–	
Asia‐Pacific region	2012–2015	896 clinical isolates of *P. aeruginosa*	–	–	–	–	100.0%	–	–	60
			–	–	–	–	14.8% MDR	–	–	
U.S.	2016	2039 clinical isolates of *P. aeruginosa*	–	–	–	–	100.0%	–	–	61
			–	–	–	–	29.5% MDR	–	–	
Iran	March to July, 2015	100 isolates of *P. aeruginosa* from wound infections of burn patients	–	–	–	–	100.00%	–	–	62
			–	–	–	–	19% MDR 7 5% XDR	–	–	
Thailand	April to December 2014	255 adult hospitalized patients with *P. aeruginosa* infections	–	–	–	–	100.0%	–	–	63
			–	–	–	–	12.5% MDR 22% XDR	–	–	

## 3 New Antibiotics Approved over the Last Five Years

Antibiotics are classified according to their modes of action, which include interference with cell wall, DNA or RNA synthesis, lysis of the bacterial membrane, inhibition of protein synthesis, and inhibition of metabolic pathways. ESKAPE bacteria exhibit an extensive range of antimicrobial resistance mechanisms, including enzymatic inactivation, target modification, cell permeability alteration, efflux pumps expression, and mechanical protection provided by biofilm formation.^21^ Widespread bacterial resistance to conventional antibiotics has revived scientific interest in identifying novel anti‐infective and pathogen clearance strategies.^64^ Some promising antibiotics that demonstrate ex vivo potential in combating ESCAPE pathogens are in the process of development for potential clinical usage.^65^

Glycopeptide antibiotics which can inhibit bacterium peptidoglycan synthesis are drugs of the last resort in the combat against drug‐resistant bacteria. Prior to the turn of the century, as the first generation of glycopeptides, vancomycin has been the mainstream therapeutic agent against serious Gram‐positive infections.^66^ Second generation glycopeptides (dalbavancin, oritavancin, and telavancin) are semisynthetic derivatives with superior pharmacokinetic and target engagement profiles that target vancomycin‐resistant infections. Dalbavancin and oritavancin demonstrate efficacy and safety that are comparable to standard care in the treatment of methicillin‐resistant *Staphylococcus aureus* (MRSA) infections.^67^ Oritavancin was clinically approved in 2014 for treatment of Gram‐positive bacteria‐associated “acute bacterial skin and soft tissue structure infections” (ABSSSI) in adults.^68^ Dalbavancin and oritavancin even represented an advance in less cost compared with standard care under baseline assumptions and scenarios.^67^

Oxazolidinone antibiotics (e.g., linezolid) inhibit protein synthesis via binding to the 50S ribosome in a broad spectrum of Gram‐positive bacteria, including MRSA, vancomycin‐resistant *S. aureus*, vancomycin‐resistant enterococci (VRE), penicillin‐resistant pneumococci, and anaerobes. Tedizolid is a second generation oxazolidinone with less adverse effects and higher potency against resistant bacterial strains than its predecessor linezolid.^69^ Tedizolid was approved by the U.S. Food and Drug Administration (FDA) in 2014 for the treatment of ABSSSI caused by MRSA.^70^ Tedizolid is superior to vancomycin in terms of clinical response.^71^ A pooled analysis of two completed Phase III clinical trials (NCT01170221 and NCT01421511; ClinicalTrials.gov registry) conducted on tedizolid for treating ABSSSI reported that a shorter 6 d treatment using tedizolid was as effacious as a 10 d treatment with linezolid.^72^ Another radomized double‐blind trial comparing the intravenous use of linezolid (600 mg every 12 h for 10 d) and tedizolid (200 mg daily for 7 d) in treating Gram‐positive noscomial infections was completed in June, 2018 (NCT02019420), although the results have not yet been published. However, high cost is an obvious drawback of tedizolid.^73^

Fluoroquinolones directly target DNA gyrase and/or topoisomerase IV that are essential in DNA replication. Ciprofloxacin has been the most widely used fluoroquinolone for treating infections caused by Gram‐negative bacteria. With increasing resistance to ciprofloxacin, FDA approved the newest fluoroquinolone delafloxacin in 2017, for the treatment of complicated ABSSSI. Delafloxacin is active in fighting against many resistant strains due to increased intracellular penetration and enhanced antibacterial activity under acidic conditions.^74^ Delafloxacin has a favorable adverse event profile in the treatment of MRSA infections compared with combined use of vancomycin and aztreonam.^75^ Current phase III clinical trial against community‐acquired pneumonia will help delineate the therapeutic role of delafloxacin.^76^ Fluoroquinolone antibiotics are approved to treat certain bacterial infections and have been used for several decades. However, in 2018, FDA reinforces safety information about serious low blood sugar levels, mental health side effects, and aortic dissections with fluoroquinolone antibiotics.^77^

Finafloxacin is another fluoroquinolone that exhibits optimum efficacy to bacterial infections in slightly acidic environments, including urinary tract infections (UTIs) and *Helicobacter pylori* infections.^78^ It was approved by the FDA as a topical suspension for the treatment of acute otitis externa caused by *P. aeruginosa* and *S. aureus*, based on the favorable results derived from two Phase III clinical trials (NCT01535560 and NCT01535599).^79^ In addition, two Phase II clinical trials have been completed which compared the efficacy/safety profiles of finafloxacin and ciprofloxacin in patients with complicated UTI and/or acute pyelonephritis (NCT00722735 and NCT01928433).^80^ Finafloxacin exhibits the early and rapid activity against resistant strains. A 5 d regimen of finafloxacin can improve microbiological eradication and clinical outcome rates, compared with the administration of ciprofloxacin for 10 d.

Ozenoxacin, the first nonfluorinated quinolone topical antimicrobial agent, is characterized by simultaneous affinity for DNA gyrase and topoisomerase IV. FDA approval of ozenoxacin in 2017 was based on evaluating the efficacy, safety, and tolerability of topical ozenoxacin in patients two months and older compared with placebo (NCT02090764,^81^ NCT01397461 and NCT02090764^82^). Ozenoxacin has demonstrated efficacy in treating impetigo complicated by the development of antimicrobial resistance, especially *S. aureus*.^83^ However, it seems ozenoxacin is unlikely to be widely used in the treatment of impetigo because of the high cost compared with current recommended treatment therapies.^84^ Nemonoxacin, another nonfluorinated quinolone, is under development for infections related to MRSA and vancomycin‐resistant pathogens. Studies of nemonoxacin for community‐acquired pneumonia (CAP) and diabetic foot infection treating have been registered. Nemonoxacin is well‐tolerated in patients with CAP.^85^ A Phase III, multicenter, randomized controlled trial showed that nemonoxacin is noninferior to levofloxacin in adult CAP patients (NCT01529476).^86^ Oral administration of nemonoxacin has been approved for the treatment of CAP in Taiwan.^87^

Plazomicin is a novel semisynthetic aminoglycoside that inhibits bacterial protein synthesis. The antibiotic is engineered to be resistant to aminoglycoside‐modifying enzymes.^88^ It was approved by the FDA in 2018 for using in adults with complicated UTI, including pyelonephritis. Plazomicin is comparable to meropenem^89^ and superior to colistin^90^ for the management of extended spectrum β‐lactamase‐producing and carbapenem‐resistant *Enterobacteriaceae* infections. Dosage reductions and therapeutic drug monitoring are required due to the limited efficacy and safety data for plazomicin. In particular, plazomicin is not recommended in patients with severe renal impairment.^89^

Eravacycline is a fully synthetic fluorocycline, which is a fourth‐generation tetracycline with the ability to inhibit protein synthesis. Eravacycline was shown to be noninferior to ertapenem and did not meet the noninferiority criteria in comparison to levofloxacin.^91^ Given its broad spectrum of activities in complicated intra‐abdominal infections, including *Enterobacteriaceae* (e.g., *Klebsiella pneumoniae* and *Escherichia coli*) and MRSA, the antibiotic was approved by Europe and USA for intravenous use in 2018.^92^

Omadacycline is an aminomethylcycline antibiotic that circumvents common tetracycline resistance mechanisms (efflux pumps and ribosomal protection proteins).^93^ Omadacycline is approved by the FDA in 2018 for the treatment of infections caused by carbapenem‐resistant *Enterobacteriaceae* and *Acinetobacter* species.^94^ In vitro omadacycline application has potent activity against Gram‐positive aerobic bacteria, including MRSA and VRE.^95^ Omadacycline is comparable to linezolid for the treatment of ABSSSI,^96^ and to moxifloxacin for the treatment of bacteria‐induced CAP in adults.^97^ The antibiotic is more active than doxycycline and minocycline against *Enterobacteriaceae* and *A. baumannii*.^98^ These results prompted omadacycline to be used for the treatment of acute bacterial skin and skin‐structure infections and CAP.

## 4 Antiresistance Potentiators

A crucial strategy to restore the antibacterial activity of current available antibiotics against multidrug‐resistant strains is the discovery of antiresistance drugs.^99^ These drugs could either block predominant bacterial resistance mechanisms or enhance the antimicrobial action of an antibiotic.^100^ Two antiresistance drugs, β‐lactamase inhibitors and efflux pump inhibitors, will be discussed.

### 4.1 β‐Lactamase Inhibitors

**Figure 2:**
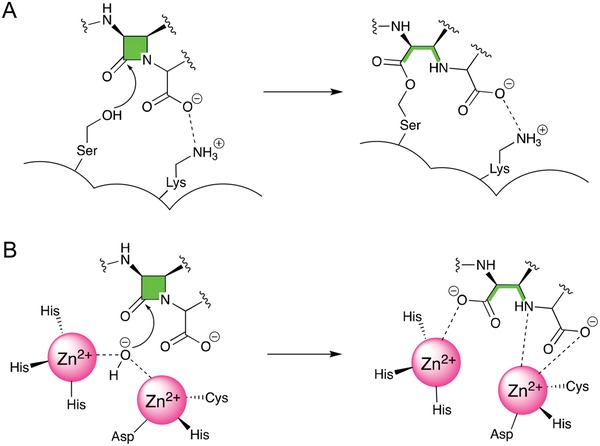
Ambler classification of β‐lactamases. A) Class A (extended spectrum), C (AmpC enzymes), and D (oxacillinase family): serine β‐lactamases‐mediated inactivation involves the attack of a nucleophilic serine. B) Class B (metallo): β‐lactam inactivation mediated by metallo‐β‐lactamases is facilitated by a nucleophilic attack via an activated water molecule coordinated to zinc ions. Reproduced with permission.^103^ Copyright 2010, Royal Society of Chemistry.

Orchestration between cell‐wall synthesis and remodeling is important for the viability of bacteria and attractive for the design of antibiotic structures.^101^ β‐lactam is the most widely used class of cell‐wall‐targeting antibiotics since the 1920s. β‐lactamase inhibitors, identified in the 1970s, are the most successful and clinically used antibiotic adjuvants to overcome resistance to β‐lactam antibiotics.^102^ β‐lactamase hydrolyzes the β‐lactam core, which is essential for antibiotic action via two molecular mechanisms: hydrolysis of enzymes that utilize an active site serine residue (class A, C, and D) or Zn^2+^ atoms (class B) to capture the antibiotic (**Figure** **2**).^103^ There is an urgent need to develop effective β‐lactamase inhibitors because no inhibitor is currently available for combating clinically challenging species that develop resistance against contemporary β‐lactam antibiotics. A deeper understanding of the surface features,^104^ resistance phenotypes,^105^ and regulation mechanisms^106^ of these enzymes will facilitate identification of potential inhibitors for therapeutic intervention.

The different generations of β‐lactamase inhibitors may be chronologically classified as: a) clavulanic acids, b) penicillin‐based sulfones, c) diazabicyclooctanes (DBOs), and d) boronic acids. Each generation of β‐lactamase inhibitors and its antibiotic representatives represents a breakthrough in the field with respect to the mechanism of inhibition and/or spectrum of activity.^102^, ^104^, ^107^ β‐lactamase inhibitors and their analogs have already demonstrated their potential as antibiotic adjuvants against MDR bacteria. Recently reported β‐lactamase inhibitors will be discussed herein.

ETX2514, designed with a modified DBO scaffold, exhibits antibacterial activity against CRE, MDR *P. aerugi*nosa and *A. baumannii*.^108^ Notably, the sulbactam–ETX2514 combination demonstrates antibacterial efficacy against clinical isolates of MDR *A. baumannii* in murine infection models, with excellent preclinical safety^108^ and low frequency of spontaneous resistance.^109^ These preliminary results are indicative of the potency of this drug combination in expanding the thin pipeline of treatment modalities for treatment of *A. baumannii*‐associated infections.

Ceftazidime‐avibactam, consisting of a synthetic β‐lactamase inhibitor (avibactam) and a third‐generation cephalosporin (ceftazidime), exhibits broad‐spectrum activity against some ESKAPE pathogens such as *P. aeruginosa*, *K. pneumonia*, and *Enterobacteriaceae*.^110^ Both ceftazidime‐avibactam and ceftolozane‐tazobactam are β‐lactam/β‐lactamase inhibitor combinations approved by the FDA for treating complicated infections.^111^ Both combinations demonstrate potent activities against *Enterobacteriaceae* and *P. aeruginosa* strains collected from various sources.^112^ Nevertheless, risk of resistance may be anticipated for both agents^113^ due to the impaired inhibition of avibactam^114^ or metallo‐β‐lactamases.^115^ Hence, accurate susceptibility testing and searching for newer alternatives should be the future direction.^116^ Relebactam and vaborbactam are β‐lactamase inhibitors. Both imipenem‐relebactam and meropenem‐vaborbactam display potential roles in infections caused by bacteria that produce extended‐spectrum β‐lactamases, *K. pneumoniae* carbapenemases, and class C β‐lactamases. A phase III clinical trial has reported the superiority of meropenem‐vaborbactam over piperacillin‐tazobactam among patients with complicated urinary tract infections, such as acute pyelonephritis.^117^ Preexisting resistance is also a common issue for combination strategy, which requires specialized prescription that is dependent upon the local hospital antibiogram (a periodic summary of antimicrobial susceptibilities of bacterial isolates from the hospital's clinical microbiology laboratory).^118^

WCK 5222 (cefepime/zidebactam) comprises a β‐lactamase inhibitor (zidebactam) and a fourth‐generation cephalosporin (cefepime). This combination demonstrates potent in vitro antimicrobial activity against a large worldwide collection of clinical isolates of *Enterobacteriaceae* and *P. aeruginosa*.^119^ Notably, the efficacy of human‐simulated WCK 5222 exposure against carbapenem‐resistant *A. baumannii* has been determined in a murine model.^120^ In addition, tolerability and safety have been observed in healthy adult subjects after intravenous administration of this drug combination.^121^ These results support further development of this drug combination for treatment of Gram‐negative bacterial infections.

### 4.2 Efflux Pump Inhibitors (EPIs)

Efflux pumps effectively exclude or reduce the intracellular concentration of antibiotics, making the pathogens significantly resistant to antibiotics. They function as a key part of the armory of ESKAPE pathogens.^122^ Efflux pumps are of considerable interest for the development of novel adjunct therapies. Potent efflux pump inhibitors may be used to reduce the prevalence of MDR bacteria and increase the efficacy of existing antibiotics.^123^ Through competitive inhibition of antibiotics on the efflux pumps of *P. Aeruginosa*, a series of pyridopyrimidine compounds such as phenylalanine arginyl β‐naphthylamide and other derivatives have been introduced as EPIs.^124^ A pyranopyridine EPI, MBX2319, was reported with better activity against *Enterobacteriaceae* than *P. aeruginosa*.^125^ Several potent EPIs were optimized in preclinical development programs, however, none of these compounds have been tested in the clinic.^122^ The impact of evolutionary selection also provides a critical context to the development of efflux pump‐targeting treatments. Recently, the AcrAB‐TolC multidrug efflux pump in *E. coli* was identified to preserve resistance acquisition through plasmid transfer.^126^ Furture understanding of the role of multidrug efflux complexs in ESKAPE is required.

### 4.3 Antivirulence Strategies

Addressing the threat of antibiotic resistance requires expanding agents to reduce selective pressures. Pathogens deploy an arsenal of virulence factors which are essential for host infection and persistence. Antivirulence therapeutic strategies target and interfere with crucial pathogenicity factors or virulence‐associated traits of the bacteria without killing or inhibiting their growth.^127^ Their application may reduce the use of broad‐spectrum antimicrobials and dampen the frequency with which resistant strains emerge.

### 4.4 Adherence, Colonization, and Invasion Inhibitors

Upon entering the host, bacterial pathogens have to travel to their respective sites of infection to initialize the disease process. Understanding the regulatory systems governing bacterial adhesion and colonization is essential for the success of antivirulence strategies. A good example is the use of pili by the uropathogenic *E. coli* for adherence, which provides potential drug targets for treating UTI.^128^ Recent reports on the development of mannosides and pilicides as antivirulence strategies provide evidence for disruption of attachment of *E. coli* to host cells.^129^ Because successful survival of pathogens requires species‐specific surface proteins,^130^ these molecules have also been investigated as therapeutic targets for *K. pneumonia*,^131^ *A. baumannii*,^132^ and *P. aeruginosa*.^133^ Nevertheless, the susceptibility to genetic changes in both microenvironment and species may create difficulties in identifying potentially effective adherence inhibitors.^134^

### 4.5 Prevention of Quorum Sensing (QS) and Biofilm Formation

**Figure 3:**
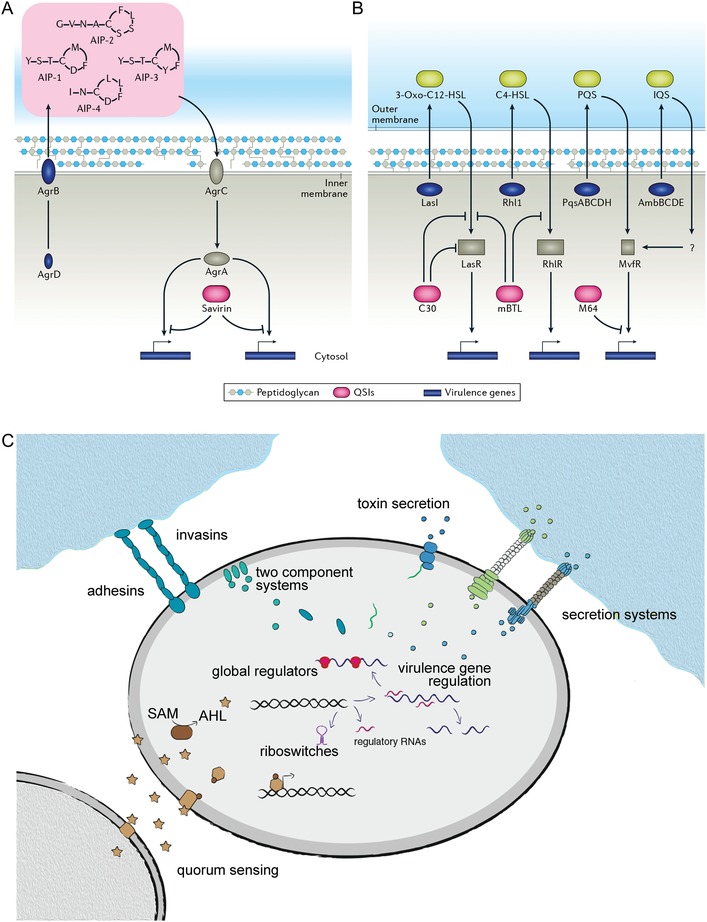
Quorum‐sensing inhibition and antivirulence strategies. A) Quorum‐sensing and inhibition mechanisms in Gram‐positive bacteria, using *S. aureus* as a model pathogen. B) Quorum‐sensing and inhibition mechanisms in Gram‐negative bacteria, using *P. aeruginosa* as a model pathogen. Synthases and exporters (dark blue) produce autoinducers that signal through receptors (gray). Activated receptors modulate gene expression of many virulence factors. Quorum‐sensing inhibitors can block ligand binding, promote receptor degradation, or block promoter binding. Quorum‐sensing feedback loops and crosstalk between pathways are omitted for simplicity. Reproduced with permission.^127^ Copyright 2017, Springer Nature. C) Summary of the targets of contemporary antivirulence strategies against Gram‐negative bacteria. These targets include (i) classical virulence factors such as adhesins/invasins, (ii) pathogen‐induced host signaling disruption by toxins, effectors, and immune modulators, (iii) microbial signal transduction and regulation, (iv) functions required for bacterial survival/persistence during infection. Reproduced with permission.^156^ Copyright 2015, Springer Nature.

Biofilm consists of a bacterial colony embedded in a complex matrix of extracellular substances, which protects the microbes from adverse environmental conditions. Compared with planktonic organisms, biofilms demonstrate increased antimicrobial resistance and result in persistent infection in clinical settings.^135^ One of the most important features of microbial biofilms is their intrinsic antibiotic tolerance. For example, *P. aeruginosa* is able to survive antibiotic treatment because of its capability to form biofilms which display both intrinsic tolerance and mutational resistance. Biofilm formation and dispersal are highly controlled processes regulated at the genetic level and by environmental signals.^136^ The main regulators of bacterial biofilms are QS systems (**Figure** **3**A,B).

Quorum sensing is a cell‐to‐cell communication process that enables bacteria to orchestrate behavior as a group and survive environmental stresses by coordinating cell‐density‐dependent gene expression.^137^ The use of QS inhibitors has been proposed as an attractive approach to prevent biofilm formation and reduce pathogenicity.^138^ Different QS systems have been identified in ESKAPE pathogens.^139^ For example, *P. aeruginosa* uses a complex QS network,^140^ which contains several possible targets^141^ and hierarchically arranges the expression of bacterial virulence genes.^142^ Identification of these QS pathways is instrumental in the design of the antibacterial agents against biofilms. Several natural and chemically synthesized QS inhibitors have been prepared for drug development. They include meta‐bromo‐thiolactone,^143^ homoserine lactone analogs and derivatives,^144^ the *Pseudomonas* quinolone signal path‐blockers,^145^ eugenol,^146^ furanone compounds,^147^ aspirin and ibuprofen,^148^ ZnO nanoparticles,^149^ and intaconimides.^150^ The activities of novel agonists and antagonists of QS have been evaluated for their reciprocal tuning activities to block pathogenesis.^151^ Materials with long‐term release of bioactive QS inhibitors may attenuate bacterial virulence and biofilm formation in many important antibacterial applications.^152^ Nevertheless, there has been substantial experimental evidence challenging the validity of QS inhibitors in combating *P. aeruginosa*.^153^ Evidence is accumulating that bacteria may develop resistance to QS inhibitors.^154^

### 4.6 Inhibition of Virulence Gene Expression

Gram‐negative bacteria possess protein secretion systems, which are molecular nanomachines spanning the two bacterial membranes to release virulence factors into the environment or direct translocation into the host cell cytosol.^155^ Recent advances in the understanding of virulence regulation have identified many control circuits and networks, including bacterial sensory and signal transduction molecules, global and specific transcriptional regulators, and RNA‐based regulatory mechanisms.^156^ Ubiquitous signaling pathways that play different roles in bacterial virulence mechanisms have become promising new targets for drug development (Figure 3C).

Two‐component systems (TCS), which most bacteria rely heavily on communication in a wide range of environmental niches, are typically composed of a sensor histidine kinase for receiving external input signals.^157^ More than 50% of TCS in *P. aeruginosa* are implicated in controlling virulence or virulence‐related behavior.^158^ A benzothiazole‐based HK inhibitor, PA14, has been applied to *P. aeruginosa* isolates derived from burn wounds and demonstrates significant attenuation in virulence behavior.^159^ *P. aeruginosa* T3SS is also critical for delivery of toxins to host cells. Screening studies have identified cyclic di‐GMP inhibitors that reduce *P. aeruginosa* biofilm formation.^160^ Other elaborate multiple virulence factors exhibited by *P. aeruginosa*, including exotoxin A (638 amino acids); a set of chemical entities have been designed to target exotoxin virulence factors.^161^ Most strains of *S. aureus* produce five different pore‐forming bicomponent leukocidins that target phagocytes and α‐hemolysin to form heptameric channels that result in cell lysis in the host.^162^ Bacterial functional membrane microdomains (FMMs) are suitable therapeutic strategies against multidrug‐resistant pathogens because they function as both barrier and exchanger. Sterol synthesis inhibitory drugs have been investigated as effective anti‐FMM drugs.^163^

The past years have seen increasing interest in adopting the antivirulence approach for developing inhibitors against bacterial kinases and other post‐translational modified enzymes, which can facilitate their survival in the host during infection.^164^ A plethora of new candidate compounds identified and validated in vitro and in vivo offers exciting prospects for the future but also constitutes a major challenge in the field.^165^ Intracellular “caseinolytic mitochondrial matrix peptidase proteolytic subunit” (ClpP) protease‐induced proteolysis is a highly conserved biological process among eubacteria. The ClpP is of pivotal importance for both the survival and virulence of pathogenic bacteria during host infection. In *S. aureus*, inactivation of ClpP renders the bacterium avirulent, which demonstrates the regulatory role of proteolysis in virulence.^166^ Deregulation of ClpP activity represents a general target for both antibiotics and antivirulence therapeutics. This spurs the development of small molecules aiming at modulating ClpP protease activity either through overactivation or inhibition.^167^ Recently, M21 has been identified from a chemical library as a noncompetitive inhibitor of ClpP. M21 attenuates *S. aureus* virulence in a mouse model, suggesting ClpP regulation as a novel drug option for controlling pathogenic bacteria.^168^ Bacterial ClpP protease is not essential in most pathogens and the majority of studies on ClpP are focused on Gram‐positive bacteria and mycobacteria. However, the same lack of essentiality and the potential to disarm pathogens without killing them renders ClpP an attractive antivirulence target for avoiding evolutionary selection pressure.^169^ Comprehensive studies on ClpP functioning in different species should be considered for expediting the development of antimicrobial agents. In a neutrophil‐depleted zebrafish model, virulence of the clpP mutant was restored in *S. aureus*, suggesting the association betweent the ClpP mechanisms and neutrophil immunity.^170^

Despite the large pool of potential antivirulence therapeutics discovered, limited clinical trials have been conducted on the use of virulence inhibitors. Antivirulence is a very attractive but incipient concept. Further research is required to demonstrate the bioavailability and pharmacodynamics of these potentially promising compounds.^171^ Combination therapy with antibiotics may be an important therapeutic tool as virulence factors are important considerations in chronic infections.^172^

## 5 Antimicrobial Peptides

AMPs, also known as host defense peptides, are important components of the innate immunological defense system. They are expressed by the host to defend against invading pathogens and boost immune response in most living species. These short cationic peptides physically consist of basic amino acids and hydrophobic residues, forming a unique water‐soluble, positively charged, and hydrophobic structure. Based on their diverse structure, AMPs are classified into α‐helical, β‐sheet, and extended peptides families.^173^, ^174^, ^175^ More than 3000 AMPs, including natural and synthesized compounds, have been isolated and characterized according to the Antimicrobial Peptide Database (https://aps.unmc.edu/AP/main.php).^176^ Many AMPs are currently being tested as candidates for developing novel antibiotics or, at least, as complements to antibiotics for treating infectious diseases.^177^ Because AMPs exhibit a broad range of antimicrobial properties^178^ and their pharmacodynamics and mutagenicity are different from antibiotics,^179^ they are potential useful for reducing the emergence of bacterial resistance.

### 5.1 Mechanisms of Action

**Figure 4:**
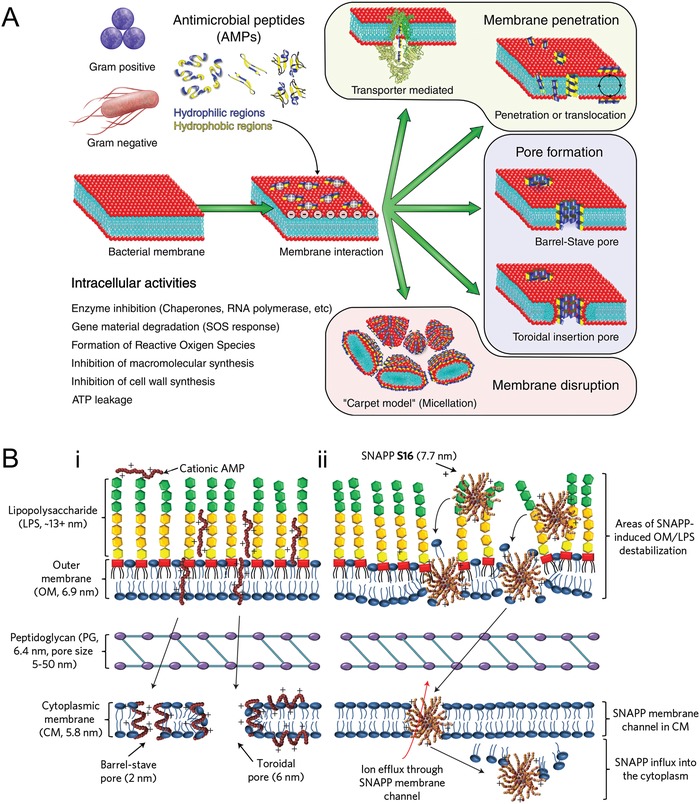
Antimicrobial peptides (AMPs). A) Mechanisms of action of antimicrobial peptides. Reproduced with permission.^177^ Copyright 2017, Elsevier. B) Comparison between the antimicrobial mechanism(s) of (i) typical membrane‐disrupting cationic AMPs and (ii) structurally nanoengineered antimicrobial peptide polymers (SNAPPs) against Gram‐negative bacteria. Reproduced with permission.^226^ Copyright 2016, Springer Nature.

The importance of AMPs resides in their multiple mechanisms of killing.^180^ The primary antimicrobial mechanism of AMPs is the disruption of bacterial membranes (**Figure** **4**A). Cationic AMPs can adhere to negatively charged bacterial membrane lipids by electrostatic interaction and kill bacteria via membrane perturbation. The selective action of these peptides is attributed to the fundamental surface differences between microbes and mammalian cells. Membrane permeabilization is generally accepted as the initial antimicrobial effect, which is important for membrane dysfunctioning, bacterial cell penetrating, and intracellular molecules targeting. Several models have been proposed to explain how AMPs induce membrane permeabilization: the barrel‐stave pore model, the theoroidal pore model, the carpet model, and other less well‐known models. It is increasingly recognized that certain AMPs inactivate bacteria without extensive membrane‐permeabilization (nonlytic) action.^181^ Other antimicrobial mechanisms of AMPs include crosstalk between innate and adaptive immunity^182^ and withdrawal of essential metal ions.^183^ Studies have shown that AMPs^184^ and their combinations with antibiotics^185^ also display anti‐biofilm properties.

Much progress has been made in peptide development and in unveiling the various characteristics of AMPs. For example, AMPs appear to be promising therapeutic options for the treatment of skin and soft tissue infections. They display dual bioactivity, namely, their propensity to control both infection and inflammation/killing activities against bacteria and immunomodulatory properties.^186^ Serious skin barrier disruption is accompanied with a high risk of MDR infection, such as MRSA,^187^ which prolongs the inflammatory phase of wound healing. Antimicrobial peptides facilitate wound recovery because of their broad spectrum of antimicrobial activity and anti‐inflammatory effect. This is particularly beneficial for the healing of chronic nonhealing wounds.^188^

However, many of the aforementioned functional observations were conducted in highly artificial in vitro systems. It remains debatable whether AMPs are really antimicrobial in vivo because isolated cell systems cannot reflect the complexity of the innate immune response.^173^ Despite the great efforts devoted to designing new peptides with improved properties,^189^ only a few AMPs have been introduced to the market or tested in clinical trials. Future studies of AMPs should focus on application‐oriented antimicrobials.

### 5.2 Antimicrobial Peptides as Drug Candidates

Peptide drugs often contain cyclic functionalities to provide additional conformational constraints and prevent proteolysis by exopeptidases. Cyclic AMPs available for clinical use include polymyxins, gramicidin, tyrothricin, bacitracin, and daptomycin.^178^ An encouraging example of AMPs introduced to the market is the polymyxins. The clinically used polymyxins, polymyxin B, and colistin are small lipopeptide molecules.^190^ Currently, they have become the last‐line treatment for infections caused by MDR *P. aeruginosa, A. baumannii*, and *K. pneumoniae*.^191^ The next‐generation polymyxins are designed to improve therapeutic index.^192^ Murepavadin is a 14‐amino‐acid cyclic peptide that represents the first member of a novel class of outer membrane protein‐targeting antibiotics.^193^ Murepavadin exhibits potent activity against a large global collection of clinically relevant XRD *P. aeruginosa*. However, murepavadin was announced to halt the Phase III trials in patients with nosocomial pneumonia due to higher acute kidney injury incidences on May 9, 2019.^194^

Macrocyclic peptide‐based scaffolds are inspirational toward the discovery of preclinical antibiotics against Gram‐negative ESKAPE pathogens.^195^ Arylomycins are a class of macrocyclic lipopeptides that inhibit bacterial type I signal peptidase. G0775 is a synthetic arylomycin derivative with potent in vitro and in vivo efficacy against Gram‐negative ESKAPE pathogens via an atypical mechanism of penetrating bacterial outer membrane. Optimized arylomycin analogs may represent a new class of Gram‐negative antibiotics.^196^

Many mammalian AMPs are currently under consideration for their functional roles in the post‐antibiotic era.^197^ Among those involved in clinical trials are cathelicidins and defensins.^198^ Defensins and cathelicidin LL‐37 are host‐defense peptides expressed and utilized by neutrophils and epithelial cells. They display antimicrobial activity against both Gram‐positive and Gram‐negative bacteria.^199^, ^200^ Defensins are composed of an α‐helical domain and two‐stranded antiparallel β‐sheet domains, while LL‐37 is a linear peptide with an amphipathic α‐helical structure.^201^ Rhesus theta‐defensin‐1 showed antimicrobial activities against MRSA and *P. aeruginosa* relevant to cystic fibrosis. This defensin demonstrates antimicrobial and immunomodulatory effects when formulated as an aerosol for treating infected lungs with cystic fibrosis in a murine model.^202^

Although publications of animal studies have increased recently, only a small group of AMPs are currently tested as pharmaceutical drugs in clinical trials.^203^ Natural AMPs often display limited efficacy in vivo; this loss of activity is largely due to systemic toxicity and their susceptibility to proteolysis.^204^ These limitations may be overcome by optimization in structural design, as shown by studies of engineered peptides. Future research should be geared toward the evaluation of AMP derivatives, analogs, or mimetics with antimicrobial activities and their feasibility of synthesis.^200^ Growing acceptance of modified peptides, residue‐ and site‐specific installation of unnatural amino acids produces a new generation of AMPs with improved pharmacological properties, particularly those with reduced proteolytic susceptibility.^205^ These unnatural amino acid‐containing AMPs exhibit a diversity of in vitro inhibitory activities against ESKAPE pathogens.^206^

Bacteriocins are ribosomally synthesized AMPs, traditionally used for food preservation. The combination of bacteriocins with other existing antibiotics may have value in clinical applications.^207^ Bacteriocins are found to regulate QS;^208^ the results suggest novel applications of bioengineered bacteriocins for targeting specific pathogens and biofilm formers.^209^ Lantibiotics, belonging to class I bacteriocins, are post‐translational peptides characterized by unusual amino acids. A number of encouraging studies have shown that lantibiotics are potential candidates against Gram‐positive bacteria, including MRSA, vancomycin intermediate *S*. *aureus* (VISA), and vancomycin‐resistant *E*. *faecalis*.^210^ To date, three lantibiotics, NAI‐107,^211^ Mu114012,^212^ and NVB302^213^ have undergone preclinical development as therapeutic agents. Incorporation of bromine into NAI‐107 produces NAI‐108 with similar antibacterial properties; its synergism with polymyxin appears to provide additional therapeutic benefits against *A. baumannii*, *K. pneumoniae*, and *P. aeruginosa*.^214^ The spectrum of antimicrobial activities may be further augmented via nanoengineering approaches or nisin modifications.^215^, ^216^ However, the impact of these modifications on the expression and antimicrobial properties of lantibiotics should be carefully characterized prior to their potential application.

Antimicrobial polymers with cationic and hydrophobic moieties are synthetic analogs of AMPs which exhibit potential against some ESKAPE pathogens. Their structure‐dependent antibacterial activity is attributed to the flexible framework, which provides opportunities for chemical pharmacophore modification and adaptation.^217^ For example, biodegradable guanidinium‐functionalized polycarbonate, a synthetic macromolecule, demonstrates high efficacy in treating MDR *A. baumannii*, *E. coli*, *K. pneumoniae*, *P. aeruginosa*, and MRSA infections in a murine model, mitigating drug resistance with negligible toxicity.^218^

Engineered cationic AMPs are synthetic peptides designed based on the sequences and structures of natural AMP.^219^ Because of its unique amphipathic structure and hydrophobic characteristics, human cathelicidin LL‐37 has been engineered to create selective and stable compounds that possess potent antimicrobial activities against ESKAPE pathogens.^220^ WLBU2 and WR12, two de novo cationic AMPs with idealized amphipathic structure, significantly enhance in vitro antimicrobial activities against clinical isolates of ESKAPE pathogens when compared to colistin and LL37.^221^, ^222^ For potential treatment of *P. aeruginosa*, WLBU2 has been used to disrupt biofilms formed on the airway epithelium without negative effects on human airway epithelial cells.^223^ WLBU2 displays superiority in efficacy over LL37 when applied via intratracheal instillation in a murine pneumonia model.^224^ WLBU2 also displays comparable activity between *S. aureus* planktonic cells and biofilms in an in vivo animal peri‐prosthetic joint infection model.^225^ Hence, cationic AMPs with optimized structures warrant further investigations on their in vivo efficacy in biofilm‐associated infections.

The termed “structurally nanoengineered antimicrobial peptide polymers” (SNAPPs) refers to star‐shaped peptide polymer nanoparticles consisting of lysine and valine residues (Figure 4B). Unlike self‐assembled antimicrobial macromolecules that rapidly dissociate, SNAPPs possess stable unimolecular architectures to maintain their concentrations. Some SNAPPs demonstrate potential as a new class of antimicrobial agents with improved therapeutic indices.^226^ However, reduction in antimicrobial activity was observed when testing was conducted using media containing simulated body fluid and animal serum. This limitation calls for the design of more efficient peptide‐based antimicrobial agents with uncompromised potency under physiological conditions.^227^ Increasing the arm number and length of SNAPPs enhance their antimicrobial activities. However, the toxicity of these compounds is concomitantly augmented. Nevertheless, the 4‐arm and 16‐arm SNAPPs with the most optimal biological activity produce no systemic damage in a murine model.^228^

Coupled with recent advances in monoclonal antibody (mAb) engineering and production capabilities, antibacterial mAbs represent a renewed opportunity in the battle against antibiotic resistance. The specificity of mAbs will unlikely cause cross‐resistance between small molecule antimicrobials and antibacterial mAbs, and harm the beneficial microbiome.^229^ In addition, mAbs exhibit long biological half‐lives. This may allow convenient dosing and prevent vaccine‐like prophylaxis from infection.^230^ However, the high affinity of mAbs and the involvement of the host immune system in their pharmacological actions may lead to complex and nonlinear pharmacokinetics and pharmacodynamics. Currently, there are three antibacterial mAbs products approved by the FDA, but none of them is prepared for ESKAPE pathogens. At least nine mAbs are undergoing clinical trials, five mAbs for *S. aureus*, three for *P*. aeruginosa, and one for *E. coli*.^230^ Antibacterial mAbs can fight against a variety of soluble exotoxins and recognize bacterial cell surface targets. Anti‐exotoxin mAbs can specifically bind to exotoxin molecules and cause toxin neutralization, to inhibit cytotoxic activity toward host cells. Examples include MEDI4893,^231^ ASN100,^232^ and Salvecin^233^ for *S. aureus*.

Monoclonal antibodies targeting bacterial surface epitopes are expected to increase bacterial clearance through enhancing antibody‐dependent phagocytosis, and/or complement‐mediated bactericidal activity, or via immune system‐independent bacterial killing. For example, 514G3^234^ for *S. aureus* and MEDI3902,^235^ panobacumab^236^ for *P. aeruginosa*. Recently, a novel antibody‐antibiotic conjugate, DSTA4637S,^237^ was introduced to kill intracellular *S. aureus* effectively because it is activated only after releasing in the proteolytic environment of the phagolysosome. Antigens as vaccines are also considered a potential therapeutic strategy. To date, five phase I/II clinical trials have been published during the past five years (**Table** **2**). More data are needed to support their effectiveness and security in different demographic contexts. These data provide insights into the pathogen‐specific antibacterial mAbs as an appealing therapeutic option for prophylaxis or treatment.

**Table 2:** Randomized clinical trials targeting at ESKAPE pathogens for clinical cure (published in last five years). Randomized clinical trials targeting at ESKAPE can be divided into four broad categories: (1) classic antibiotics, such as β‐lactam, fluoroquinolone, and sulfonamide antibiotics; (2) combination therapy, such as β‐lactam/aminoglycoside, β‐lactam/β‐lactamase inhibitor, and sulfonamide/dihydrofolate reductase inhibitor; (3) non‐antibiotic therapy, such as phages, probiotics, and natural agents; and (4) preventive measure, such as chlorhexidine washing, vaccine, and environment disinfection

Drug category [reference]	Intervention (administration)	ESKAPE target	Study design	Subject characteristics	Treatment groups	Duration	Major outcomes and implications
Bacteriophages^382^	Daily PP1131: a cocktail of 12 bacteriophages 1 × 10^6^ PFU mL^−1^ (topical)	*P. aeruginosa*	Phase I/II, double‐blind RCT	Patients (aged ≥18 years) with burn wounds infected with *P. aeruginosa* (*n* = 26)	PP1131 (*n* = 13), standard of care (*n* = 13)	7 d, with 14 d follow‐up	Low concentrations of PP1131 reduced bacterial burden in infected wounds, but at a slower speed than standard of care. Further studies using enhanced phage concentrations with larger sample size are warranted.
Maggot therapy^238^	*Lucilia sericata*: a dose of 5–7 maggots cm^−2^ of wound surface applied at 48 h intervals (topical)	*S. aureus* and *P. aeruginosa*	Phase II, RCT	Adult patients with diabetic foot ulcers (*n* = 50)	Treatment: maggot therapy (*n* = 25), control: conventional treatment such as antibiotic therapy, debridement, and offloading (*n* = 25)	2–4 d	Maggot therapy is a safe and efficacious treatment for diabetic foot ulcers. More evidence regarding the ability of maggots to treat abscesses in humans is needed.
Probiotics^239^	*Lactobacillus rhamnosus* HN001: 1 × 10^10^ colony forming units once daily (oral)	MRSA, MSSA, and *S. aureus*	Phase II, double‐blind RCT	Subjects with *S. aureus* colonization at several body sites (*n* = 113)	Probiotic (*n* = 52), placebo (*n* = 61)	4 weeks	Use of *Lactobacillus rhamnosus* strain reduced odds of carriage of *S. aureus* in the gastrointestinal tract. Further studies are needed to explore alternative probiotic effective on other body sites.
Lipoglycopeptide antibiotic^240^	Oritavancin: 200 mg once (i.v.)	*S. aureus*, MRSA, MSSA, and VRE *faecium*	Phase III, multicenter, double‐blind RCT	Adult patients with acute bacterial skin and skin structure infections caused by Gram‐positive pathogens (*n* = 1959)	Oritavancin (*n* = 978), vancomycin: 1 g or 15 mg kg^−1^ twice daily (*n* = 981)	>7 d	Oritavancin was noninferior to vancomycin on clinical response. Single‐dose oritavancin may offer additional flexibility in treatment.
Glycopeptide antibiotic^241^	Vancomycin: 2 g (topical)	*S. aureus*, *P. aeruginosa*, *Acinetobacter*	Phase III, RCT	Patients with elective spine surgery (*n* = 380)	Vancomycin (*n* = 193), no application of local antibiotic (*n* = 187)	Over the fascia before the final closure	Intrawound application of vancomycin was not associated with the risk of surgical site infection. Further understanding of local antibiotics is required.
Fluoroquinolone antibiotic^242^	Ciprofloxacin dry powder: twice daily 32.5 mg (inhalation)	*P. aeruginosa* and *S. aureus*	Phase III, double‐blind RCT	Patients with noncystic fibrosis bronchiectasis (*n* = 416)	Ciprofloxacin (*n* = 278), placebo (*n* = 138)	14 or 28 d on/off treatment cycles for 48 weeks	Ciprofloxacin 14 d on/off significantly prolonged time to first exacerbation and reduced the frequency of exacerbation compared with matching placebo. Ciprofloxacin dry powder for inhalation was well tolerated and has the potential to be an effective treatment option.
Fluoroquinolone antibiotic^243^	Ciprofloxacin dry powder: twice daily 32.5 mg (inhalation)	*P. aeruginosa* and *S. aureus*	Phase III, double‐blind RCT	Patients with noncystic fibrosis bronchiectasis (*n* = 521)	Ciprofloxacin (*n* = 347), placebo (*n* = 174)	14 or 28 d on/off treatment cycles for 48 weeks	Neither ciprofloxacin treatment achieved statistical significance. The extrapolation method and significance levels should take into consideration in further studies.
Fluoroquinolone antibiotic^244^	Ciprofloxacin: 20 mg kg^−1^ daily (oral)	*K. pneumonia*	Phase III, RCT	Children with acute lymphoblastic leukemia or lymphoma scheduled to undergo chemotherapy (*n* = 87)	Ciprofloxacin (*n* = 44), placebo (*n* = 43)	3 weeks	The *K. pneumoniae* susceptibility and minimal inhibitory concentrations of ceftazidime were not different between the two groups. The use of ciprofloxacin prophylaxis needs further studies.
Fluoroquinolone antibiotic^245^	Delafloxacin: twice daily 300 mg (i.v.)	MRSA, other MDR Gram‐positive and Gram‐negative bacteria	Phase III, double‐blind multicenter RCT	Patients with acute bacterial skin and skin‐structure infections (*n* = 660)	Delafloxacin (*n* = 331), vancomycin 15 mg kg^−1^ plus aztreonam 2 g (*n* = 329)	5–14 d	Delafloxacin was statistically noninferior to vancomycin‐aztreonam at 48–72 h after initiation. Delafloxacin provides an option as monotherapy in the treatment of acute bacterial skin and skin structure infections.
Fluoroquinolone antibiotic^246^	Delafloxacin: 300 mg i.v. every 12 h for 3 d followed by 450 mg oral (i.v./oral)	*S. aureus*, MRSA, other MDR Gram‐positive and Gram‐negative bacteria	Phase III, double‐blind multicenter RCT	Patients with acute bacterial skin and skin‐structure infections (*n* = 850)	Delafloxacin (*n* = 423), vancomycin 15 mg kg^−1^ plus aztreonam 2 g (*n* = 427)	5–14 d	Delafloxacin was noninferior to vancomycin–aztreonam combination therapy for both the objective response and the investigator‐assessed response. Delafloxacin was well tolerated as monotherapy in treatment of acute bacterial skin and skin structure infections.
Fluoroquinolone antibiotic^247^	Levofloxacin: 240 mg (inhalation solution)	*P. aeruginosa*	Phase III, multicenter, double‐blind RCT	Patients (aged ≥12 years) with cystic fibrosis and chronic *P. aeruginosa* infection (*n* = 330)	Levofloxacin (*n* = 220), placebo (*n* = 110)	28 d course	Levofloxacin did not show the superiority over placebo in the primary outcome of reduction in pulmonary exacerbations. Given the proven tolerability and clinical efficacy of levofloxacin, further examination is needed.
Oxazolidinone antibiotic^248^	Tedizolid: once daily 200 mg for 6 d (i.v. and optional oral)	*S. aureus and* MRSA	Phase III, double‐blind RCT	Patients with acute bacterial skin and skin‐structure infections (*n* = 666)	Tedizolid (*n* = 332), linezolid: twice daily 600 mg for 10 d (*n* = 334)	6 or 10 d	Tedizolid was noninferior to linezolid on clinical response and adverse events. Tedizolid may become a useful option.
Oxazolidinone antibiotic^249^	Tedizolid: once daily 200 mg (i.v./oral)	MRSA	Phase III, RCT	Patients with skin and soft tissue infections or related bacteremia (*n* = 125)	Tedizolid (*n* = 84), linezolid: twice daily 600 mg (*n* = 41)	7–21 d	Tedizolid treatment achieved favorable clinical/microbiological efficacy and safety profile compared with linezolid. Tedizolid may be an appropriate antibiotic.
Oxazolidinone antibiotic^495^	Linezolid: twice daily 600 mg (i.v.)	MRSA	Phase IV, multicenter RCT	Patients with nosocomial pneumonia caused by MRSA (*n* = 448)	Linezolid (*n* = 224), vancomycin: twice daily 15 mg kg^−1^ (*n* = 224)	Administered for 7–14 d, 30 d follow‐up	Linezolid showed a higher cure rate for diabetic patients with MRSA. Linezolid may be an option for diabetic patients.
Cyclic lipopeptide antibiotic^496^	Daptomycin: 7–12 mg kg^−1^ once daily according to patient age (i.v./oral)	*S. aureus*, MRSA	Phase IV, evaluator‐blinded, multicenter RCT	1‐ to 17‐year‐old patients with *S. aureus* bacteremia (*n* = 82)	Daptomycin (*n* = 55), standard‐of‐care: mainly vancomycin or cefazolin (*n* = 27)	5–42 d	Daptomycin was a safe and well‐tolerated alternative. The efficacy of daptomycin need statistical conclusion.
Cyclic lipopeptide antibiotic^497^	Daptomycin: 5–10 mg kg^−1^ once daily according to patient age (i.v./oral)	*S. aureus*, MRSA	Phase IV, evaluator‐blinded, multicenter RCT	1‐ to 17‐year‐old patients with complicated skin and skin structure infections caused by Gram‐positive pathogens (*n* = 389)	Daptomycin (*n* = 257), standard‐of‐care: primarily vancomycin, clindamycin, and penicillins (*n* = 132)	≤14 d	Daptomycin was well tolerated, safety, and efficacy. Further studies confirming daptomycin as a suitable alternative are worthwhile.
Diaminopyrimidine antibiotic^250^	Iclaprim: 80 mg every 12 h (i.v.)	MRSA, MSSA	Phase III, double‐blind multicenter RCT	Patients with acute bacterial skin and skin‐structure infections due to Gram‐positive pathogens (*n* = 1198)	Iclaprim (*n* = 593), vancomycin: 15 mg kg^−1^ every 12 h (*n* = 605)	5–14 d	Iclaprim and vancomycin were comparable for early clinical response secondary endpoints and safety. Iclaprim provides an option for treating infections caused by Gram‐positive pathogens.
Macrolide antibiotic^251^	Azithromycin: 2 g (oral)	*S. aureus*	Phase III, double‐blind RCT	Newborns (*n* = 843)	Azithromycin (*n* = 419), placebo (*n* = 424)	One dose during labor and 8 weeks follow‐up	Azithromycin decreased bacteria prevalence and infections in both women and their offspring but no significant difference was seen in the incidence of *S. aureus* related purulent conjunctivitis. Larger studies designed to evaluate the effect of azithromycin on infections are warranted.
Sulfonamide antibiotic—dihydrofolate reductase inhibitor^252^	Trimethoprim‐sulfamethoxazole: 320 mg/1600 mg twice daily (oral)	MRSA	Phase III, double‐blind RCT	Patients with a drained cutaneous abscess (*n* = 1265)	Trimethoprim‐sulfamethoxazole (*n* = 629), placebo (*n* = 636)	7 d	Trimethoprim‐sulfamethoxazole had a higher cure rate. The benefit of adjunctive antibiotic should be further explored in abscess treatment.
Lincosamide antibiotic, sulfonamide antibiotic—dihydrofolate reductase inhibitor^253^	Clindamycin: 300 mg three times daily and trimethoprim–sulfamethoxazole: 80 mg/400 mg twice daily (oral)	MRSA	Phase III, double‐blind RCT	Patients with skin abscesses treated with incision and drainage (*n* = 786)	Clindamycin (*n* = 266), trimethoprim—sulfamethoxazole (*n* = 263), placebo (*n* = 257)	10 d	The cure rate in both active treatment group was higher. These improved outcomes should be further weighed with the adverse events.
Lincosamide antibiotic, sulfonamide antibiotic—dihydrofolate reductase inhibitor^254^	Clindamycin: 300 mg four times daily or trimethoprim—sulfamethoxazole: 320 mg/1600 mg twice daily (oral)	MRSA, MSSA	Phase III, double‐blind, multicenter RCT	Patients with an uncomplicated wound infection (*n* = 401)	Clindamycin (*n* = 203), trimethoprim—sulfamethoxazole (*n* = 198)	Treatment for 7 d, 6–8 weeks follow‐up	Clindamycin had a significantly lower rate of recurrence. Further study evaluating the effect of antibiotic on recurrent infection is warranted.
Cephalosporin antibiotic^255^	Ceftaroline fosamil (i.v./oral)	MRSA	Phase II, multicenter, observer‐blinded RCT	Pediatric patients with acute bacterial skin and skin structure infections (*n* = 159)	Ceftaroline fosamil (*n* = 106), comparison: vancomycin or cefazolin, plus optional aztreonam (*n* = 53)	5–14 d	Ceftaroline was well tolerated and effective. Ceftaroline offers an alternative treatment approach via the oral route.
Cephalosporin antibiotic^256^	Ceftaroline fosamil (i.v./oral)	*S. aureus*, MRSA	Phase II, multicenter, CT	Pediatric patients with community‐acquired bacterial pneumonia (*n* = 160)	Ceftaroline (*n* = 121), ceftriaxone (*n* = 39)	5–14 d	Ceftaroline was well tolerated and effective. Powered inferential statistics are warranted.
Cephalosporin antibiotic^257^	Cefiderocol: 2 g, three times daily (i.v.)	*P. aeruginosa* and *A. baumannii*	Phase II, multicenter, double‐blind, parallel‐group RCT	Patients (≥18 years) with complicated urinary tract infection or acute uncomplicated pyelonephritis (*n* = 452)	Cefiderocol (*n* = 303), imipenem‐cilastatin: 1 g each (*n* = 149)	7–14 d	Cefiderocol was well tolerated and demonstrated noninferiority. The results will provide the basis for the approach of a new drug.
Cephalosporin antibiotic, sulfonamide antibiotic—dihydrofolate reductase inhibitor^258^	Cephalexin: 500 mg four times daily, trimethoprim‐sulfamethoxazole: 320 mg/1600 mg twice daily (oral)	MRSA	Phase III, double‐blind, multicenter RCT	Patients with uncomplicated cellulitis (*n* = 496)	Cephalexin plus trimethoprim‐sulfamethoxazole (*n* = 248), cephalexin plus placebo (*n* = 248)	7 d	Clinical resolution rate was not significantly different. Further research on combination antibiotics may be needed.
Cephalosporin antibiotic, β‐lactam/β‐lactamase inhibitor combination^259^	Cefepime: 1 g every 6 h and amoxicillin/clavulanic acid: 1.2 g every 6 h	Carbapenem‐resistant *K. pneumoniae*	Phase I, RCT	Patients with confirmed bla_KPC_‐positive *K. pneumoniae* infection (*n* = 62)	Cefepime and amoxicillin/clavulanic acid (*n* = 30), tigecycline (*n* = 32)	Treatment over 48 h, 28 d follow‐up	The mortality of the study group was tended to be lower. Cefepime and amoxicillin/clavulanic acid combination may be an effective and economical option.
Penicillin antibiotic, aminoglycoside antibiotic^260^	Benzylpenicillin: 50000 i.u. kg^−1^, gentamicin: 6 mg kg^−1^ daily (i.v.)	*S. aureus*, *K. pneumoniae*	Phase III, RCT	Infants <60 d with possible severe sepsis (*n* = 331)	Benzylpenicillin and gentamicin (*n* = 161), ceftriaxone: 50–100 mg kg^−1^ (*n* = 170)	5–14 d	Outcome from possible severe bacterial infections was similar. Further sequelae observation after hospital discharge is worthwhile.
Aminoglycoside antibiotic/monoxycarbolic acid antibiotic^261^	Gentamicin (0.1%)/mupirocin (2%) alternate regimen in month: daily application (topical)	*S. aureus*, *P. aeruginosa*	Phase II, open‐label RCT	Patients receiving peritoneal dialysis (*n* = 146)	Gentamicin/mupirocin (*n* = 75), gentamicin (*n* = 71)	A total follow‐up duration of 174 and 181 patient‐years, respectively	Alternating application showed similar preventive effect on exit‐site infection except for *P. aeruginosa*, but inferior on peritonitis. The choice of topical agents should take the spectrum of activity and potency into consideration.
Miscellaneous antibiotic, aminoglycoside antibiotic^262^	Aztreonam: 75 mg, three times daily) alternating with tobramycin: 300 mg, twice daily (inhalation solution)	*P. aeruginosa*	Phase III, multicenter, double‐blind RCT	Patients with cystic fibrosis (*n* = 90)	Aztreonam/tobramycin (*n* = 43), placebo/tobramycin (*n* = 47)	3 cycles of 28 d	Continuous alternating therapy reduced the rates of exacerbation and respiratory hospitalizations. Study enrollment was limited; thus, the additional clinical benefit was underpowered.
Aminoglycoside antibiotic^263^	Tobramycin: 112 mg, twice daily (powder for inhalation)	*P. aeruginosa*	Phase III, multicenter, double‐blind RCT	Cystic fibrosis patients aged 6–21 years chronically infected with *P. aeruginosa* (*n* = 62)	Tobramycin (*n* = 30), placebo (*n* = 32)	<7 cycles, a single treatment cycle consists of 28 d on‐treatment followed by 28 d off‐treatment	Tobramycin powder for inhalation showed safety and suppression of sputum *P. aeruginosa* density. Long‐term treatment with tobramycin may offer an option for treatment of cystic fibrosis.
β‐Lactam^264^	Meropenem: 1 g (i.v.)	*K. pneumonia*	RCT	Patients with indications for prostatic biopsy (*n* = 110)	Meropenem (*n* = 55), ciprofloxacin: 500 bid os^−1^ 3 d (*n* = 55)	15 d follow‐up	A single dose of meropenem is safe and effective. Further research to avoid possible infectious complications is warranted.
β‐Lactam^500^	Meropenem (i.v.)	*K. pneumoniae*, *P. aeruginosa*	Phase IV, RCT	Elderly patients with lower respiratory tract infections (*n* = 79)	Individualize meropenem therapy (*n* = 39), meropenem dose decided by physician (*n* = 40)	7–13 d, 1 week follow‐up	Dosing regimens based on pharmacokinetic and pharmacodynamic models improve clinical response for lower respiratory tract infections. Further study to develop individualized antibiotic regimens is warranted.
β‐Lactam/β‐lactamase inhibitor combination^265^	Piperacillin‐tazobactam: 4.5 g every 6 h (i.v.)	ESBL producing *K. pneumonia*	Phase III, open‐label, multicenter RCT	Patients with bloodstream infection and ceftriaxone resistance (*n* = 379)	Piperacillin‐tazobactam (*n* = 188), meropenem: 1 g every 8 h (*n* = 191)	4–14 d, 30 d follow‐up	Treatment with piperacillin‐tazobactam was inferior compared with meropenem. Whether alternative agents remain effective needs more evidence to support.
β‐Lactam/β‐lactamase inhibitor combination^266^	Meropenem‐vaborbactam: 2 g/2 g every 8 h (i.v.)	*K. pneumonia*	Phase III, multicenter RCT	Patients with complicated urinary tract infection (*n* = 545)	Meropenem‐vaborbactam (*n* = 272), piperacillin‐tazobactam: 4 g/0.5 g every 8 h (*n* = 273)	Total treatment 10 d, 14 d follow‐up	Meropenem‐vaborbactam was noninferior to piperacillin‐tazobactam in the overall cure rate. Further research to understand the spectrum of pathogens is warranted.
β‐Lactam/β‐lactamase inhibitor combination^267^	Ceftazidime‐avibactam: 2 g/0.5 g every 8 h (i.v.)	*K. pneumonia*, *P. aeruginosa*	Phase III, double‐blind, multicenter RCT	Patients with complicated urinary tract infection (*n* = 810)	Ceftazidime‐avibactam (*n* = 393), doripenem: 0.5 g every 8 h (*n* = 417)	10–14 d, 45–52 d follow‐up	Both treatments showed comparable efficacy against ceftazidime‐nonsusceptible pathogens. Ceftazidime‐avibactam may offer a clinical option for treating carbapenemase‐producing uropathogens.
β‐Lactam/β‐lactamase inhibitor combination^268^	Ceftazidime‐avibactam: 2 g/0.5 g every 8 h (i.v.)	*K. pneumoniae*, *P. aeruginosa*	Phase III, double‐blind, multicenter RCT	Adults with nosocomial pneumonia (*n* = 726)	Ceftazidime‐avibactam (*n* = 356), 1000 mg meropenem: 1 g every 8 h (*n* = 370)	7–14 d, 28–32 follow‐up days after randomization	Ceftazidime‐avibactam was noninferior to meropenem in the treatment of nosocomial pneumonia. These results support ceftazidime‐avibactam as a potential alternative agent to carbapenems.
β‐Lactam/β‐lactamase inhibitor combination^269^	Ceftazidime‐avibactam: 2 g/0.5 g every 8 h plus metronidazole: 0.5 g every 8 h (i.v.)	*Enterobacteriaceae* and *P. aeruginosa*	Phase III, double‐blind, multicenter RCT	Patients with complicated intra‐abdominal infection (*n* = 1043)	Ceftazidime‐avibactam plus metronidazole (*n* = 520), meropenem: 1 g every 8 h (*n* = 523)	5–14 d, 42–49 d follow‐up after randomization	Ceftazidime‐avibactam plus metronidazole was noninferior to meropenem in the treatment. Ceftazidime‐avibactam may offer a clinical option for treating ESBL‐producing organisms.
β‐Lactam/β‐lactamase inhibitor combination^270^	Ceftazidime‐avibactam: 2 g/0.5 g every 8 h (i.v.)	Ceftazidime‐resistant *Enterobacteriaceae* or *P. aeruginosa*	Phase III, multicenter, open‐label RCT	Patients with complicated urinary tract infection or complicated intra‐abdominal infection (*n* = 333)	Ceftazidime‐avibactam (*n* = 165), meropenem, imipenem, doripenem, colistin, tigecycline, and combination treatment (*n* = 168)	5–21 d of treatment, 7–10 d follow‐up	Ceftazidime‐avibactam was clinically effective as carbapenems. These promising results support the further use of ceftazidime‐avibactam in resistant Gram‐negative infections.
β‐Lactam/β‐lactamase inhibitor combination^271^	Ceftolozane‐tazobactam: 1 g/0.5 g every 8 h plus metronidazole: 0.5 g every 8 h (i.v.)	*K. pneumoniae*, *P. aeruginosa*	Phase III, double‐blind, multicenter RCT	Patients with complicated urinary tract infections and complicated intra‐abdominal infections (*n* = 806)	Ceftolozane‐tazobactam (*n* = 389), meropenem: 0.5 g every 8 h (*n* = 417)	4–10 d, 38–45 d follow‐up	Ceftolozane‐tazobactam plus metronidazole was noninferior to meropenem. The use of ceftolozane‐tazobactam in *P. aeruginosa* implicated infections required further study.
β‐Lactam/β‐lactamase inhibitor combination^272^	Ceftolozane‐tazobactam: 1 g/0.5 g every 8 h (i.v.)	*K. pneumoniae*, *P. aeruginosa*	Phase III, double‐blind, multicenter RCT	Patients with pyelonephritis and complicated urinary‐tract infections (*n* = 800)	Ceftolozane‐tazobactam (*n* = 398), levofloxacin: 750 mg once daily (*n* = 402)	7 d,	Ceftolozane‐tazobactam led to better responses than high‐dose levofloxacin. Further study using the combination for infections caused by multidrug‐resistant pathogens is worthwhile.
Chlorhexidine and monoxycarbolic acid antibiotic^273^	Active bathing to eliminate infection 4% rinse‐off chlorhexidine for bathing or showering and 2% leave‐on chlorhexidine for bed baths, wounds and devices plus 2% nasal mupirocin for MRSA carriers (topical)	MRSA, VRE	Cluster‐randomized trial	Patients in non‐critical‐care units (*n* = 339 902)	Decolonization group (*n* = 183 013), routine soap bathing care group (*n* = 156 889)	Daily bathing or showering was encouraged. Twice‐daily nasal mupirocin ointment for 5 d.	Decolonization with universal chlorhexidine bathing and targeted mupirocin for MRSA carriers did not significantly reduce multidrug‐resistant organisms in non‐critical‐care patients. Further research is needed to confirm the effect if the decolonization strategy is applied only to patients with medical devices.
Chlorhexidine and monoxycarbolic acid antibiotic^274^	Decolonization: 4% rinse‐off chlorhexidine for daily bathing or showering, 0.12% chlorhexidine mouthwash, and 2% nasal mupirocin twice daily (topical)	MRSA	Phase III, multicenter, RCT	Patients colonized with MRSA (*n* = 2121)	Decolonization group (*n* = 1058), hygiene education group (*n* = 1063)	5 d twice per month for 6 months	Postdischarge MRSA decolonization with chlorhexidine and mupirocin led to a 30% lower risk of MRSA infection than education alone in one year. Further research is needed to confirm whether the observed lower risk of infection will apply to less severe infections.
Chlorhexidine^275^	Chlorhexidine gluconate: 0.12% in oropharyngeal and 4% in nasopharyngeal four times a day (topical)	*S. aureus*	Phase III, double‐blind RCT	Adults patients scheduled for major anatomical pulmonary resection surgery (*n* = 450)	Chlorhexidine gluconate (*n* = 226), placebo (*n* = 224)	Perioperative 3 d	Chlorhexidine gluconate decontamination did not decrease the need for mechanical ventilation nor the rate of respiratory healthcare‐associated infections. Effective decontamination protocol is warranted in further studies.
Chlorhexidine^276^	Chlorhexidine: 4% daily (topical)	*S. aureus*	Phase I, RCT	Peritoneal dialysis patients (*n* = 89)	Chlorhexidine (*n* = 50), saline (*n* = 39)	6 and 12 months	The rates of *S. aureus* colonization rates were significantly lower with the intervention. Chlorhexidine care at the exit site may be a potential strategy.
Chlorhexidine^277^	Chlorhexidine digluconate: 2% twice daily (topical)	MRSA, CRAB	Phase I, double‐blind RCT	Patients undergoing mechanical ventilation (*n* = 16)	Chlorhexidine (*n* = 8), placebo: 0.9% NaCl (*n* = 8)	10 d	Chlorhexidine reduced the incidence of oral colonization by *S. aureus*. Further studies of prevention of ventilator‐associated pneumonia are needed.
Vaccine^278^	3‐Antigen *S. aureus* vaccine (vaccination)	*S. aureus*	Phase I, double‐blind RCT	Healthy volunteers aged 50–85 (*n* = 312) and 18–24 (*n* = 96)	Vaccine (*n* = 306), placebo (*n* = 102)	Single dose	Immune responses were robust in both age cohorts. Considering the safety and tolerability, the mid‐dose‐level vaccine antigens is warranted for further research.
Vaccine^279^	4‐Antigen or 3‐antigen *S. aureus* vaccine (vaccination)	*S. aureus*	Phase I/II, double‐blind RCT	Healthy adults aged 65–85 years (*n* = 283)	Vaccine (*n* = 223), placebo (*n* = 60)	Single dose	Both vaccines induced rapid and robust functional immune responses. Further studies on the *S. aureus* prophylactic vaccine are worthwhile.
Vaccine^280^	4‐Antigen *S. aureus* vaccine (vaccination)	*S. aureus*	Phase I/II, double‐blind RCT	Healthy adults aged 18–64 years (*n* = 454)	Vaccine (*n* = 342), placebo (*n* = 112)	Single dose with 12 months follow‐up	The vaccine safely induced durable immune responses. Further development of this vaccine is worthwhile.
Vaccine^281^	MEDI4893 (vaccination)	*S. aureus*	Phase I, RCT	Healthy adults aged 18–65 years (*n* = 33)	MEDI4893 (*n* = 26), placebo (*n* = 7)	Single dose with 360 d follow‐up	Administration was not associated with serious adverse events. Further development for the prevention of *S. aureus*‐related pneumonia is worthwhile.
Vaccine^282^	IC43 (vaccination)	*P. aeruginosa*	Phase II, multicenter, partially blinded RCT	ICU patients on mechanical ventilation (*n* = 401)	Vaccine 100 µg with adjuvant (*n* = 104), vaccine 100 µg without adjuvant (*n* = 98), vaccine 200 µg with adjuvant (*n* = 101), placebo (*n* = 98)	Twice in a 7 d interval and 90 d follow‐up	IC43 vaccination produced a significant immunogenic effect but the infection rates did not show significant difference. The dose and formulation need further testing of its possible benefit of improved survival.
Preventive measure^283^	Vitamin D: 4000 IU d^−1^ (oral)	MRSA	Phase I, double‐blind RCT	Persistent MRSA carriers with 25‐hydroxy vitamin D_3_ < 75 nmol L^−1^ (*n* = 65)	Vitamin D (*n* = 32), placebo (*n* = 33)	12 months	Vitamin D supplementation did not influence MRSA carriage. There is still an unmet medical need to find novel strategies to eradicate MRSA.
Preventive measure^284^	Cephalexin: 2 g (oral)	*S. aureus*	Phase II, double‐blind RCT	Patients booked for flap or graft closure on the ear and nose (*n* = 154)	Cephalexin (*n* = 77), placebo (*n* = 77)	40–60 min prior to surgery	A single high‐dose preoperative oral cephalexin significantly reduced surgical site infections. Antibiotic prophylaxis warranted in further studies.
Natural agent^285^	Honey: 30% three times d^−1^ (topical)	MRSA	Phase II, RCT	Adults patients with nasal MRSA (*n* = 100)	Honey (*n* = 50), mupirocin 2% (*n* = 50)	5 or 10 d	Medical‐grade honey showed a decolonization rate of 42.8%. Honey is a potential option for decolonization.
Terminal room disinfection^286^	Quaternary ammonium, ultraviolet light, hypochlorite (environment)	MRSA, VRE, multidrug‐resistant Acinetobacter	Multicenter, cluster‐randomized, crossover trial	Exposed patients (*n* = 21 395)	Quaternary ammonium (*n* = 4916), ultraviolet light (*n* = 5178), hypochlorite (*n* = 5438), hypochlorite and ultraviolet light (*n* = 5863)	Consecutive 7‐month study periods	Ultraviolet light reduced the environmental bioburden of target organisms. Enhanced terminal room disinfection may decrease the environmental source of pathogens.
Antiseptic solutions^287^	Polyhexamethylene biguanide: 0.1% (soaked dressings)	*S. aureus*	Phase I, double‐blind RCT	Patients with full‐thickness skin grafting (*n* = 40)	Polyhexamethylene biguanide (*n* = 20), sterile water (*n* = 20)	7 d follow‐up	No significant differences were detected between the case and control. The presence of *S. aureus* in wounds may result in more surgical site infections.
Bleach baths^288^	Bleach baths: hypochlorite (topical)	*S. aureus*	Phase I, single‐blinded RCT	Pediatric patients with atopic dermatitis (*n* = 21)	Corticosteroids+ bleach (*n* = 10), corticosteroids (*n* = 11)	4 weeks	Bleach baths may not influence the cutaneous microbiome. Further studies are needed to find the role of dilute bleach baths in the long‐term maintenance.
Adjunctive agent^289^	Nitric oxide: 10 ppm with tobramycin and ceftazidime (inhalation)	*P. aeruginosa*	Phase II, RCT	Patients (≥12 years) with cystic fibrosis and chronic *P. aeruginosa* colonization (*n* = 12)	Nitric oxide (*n* = 6), placebo with tobramycin and ceftazidime (*n* = 6)	7 d	Nitric oxide demonstrated significant reduction in *P. aeruginosa* biofilm aggregates. Potential strategies to induce biofilms disruption are worthwhile.
Adjunctive agent^290^	Rifampicin: 600 or 900 mg d^−1^ according to weight (i.v./oral)	*S. aureus*	Phase III, double‐blind, multicenter RCT	Adults with *S. aureus* bacteremia (*n* = 758)	Rifampicin (*n* = 370), placebo (*n* = 388)	2 weeks	Adjunctive rifampicin did not did not improve outcomes from *S. aureus* bacteremia. Rifampicin with standard antibiotic therapy may not provide overall benefit.
Recombinant human lactoferrin^291^	Talactoferrin: 150 mg kg^−1^ twice daily (oral/enteral)	*Enterobacter* and *Klebsiella*	Phase I/II, RCT	Infants with birth weights ranging from 750 to 1500 g (*n* = 120)	Talactoferrin (*n* = 60), placebo (*n* = 60)	28 d	Talactoferrin demonstrated influence on fecal microbiota and induction of hospital‐acquired infections. Future research for gut‐related microbiota modification is warrant.

Abbreviations: PFU, plaque‐forming units; RCT, randomized controlled trial; i.v., Intravenous; CRAB, Carbapenem‐resistant *Acinetobacter baumannii*; MSSA, Methicillin‐susceptible *S. aureus*; MRSA, Methicillin‐resistant *S. aureus*; VISA, vancomycin intermediate *S. aureus*; VRSA, vancomycin‐resistant *S. aureus*; VRE, vancomycin‐resistant *Enterococci*; ESBL, extended spectrum β lactamase; CRAB, Carbapenem‐resistant *Acinetobacter baumannii*.

### 5.3 Resistance to Antimicrobial Peptides

Antimicrobial peptides are among the newest antibiotic‐mimicking therapeutics. However, there are considerable experimental data describing the resistance mechanisms to AMPs, including membrane modification, efflux, and generating mutants.^177^, ^292^, ^293^ To reduce AMP resistance, changes in the molecular structure, modifications of biochemical characterization, and combination with common antibiotics have been reported.^293^ The aprotinin is the first inhibitor identified to be capable of inhibiting AMP resistance in multiple pathogens.^294^ More in‐depth research is required before AMPs can be recommended for clinical application.

## 6 Nanodelivery Strategies

### 6.1 Nanotechnology‐Based Drug Delivery Systems

**Figure 5:**
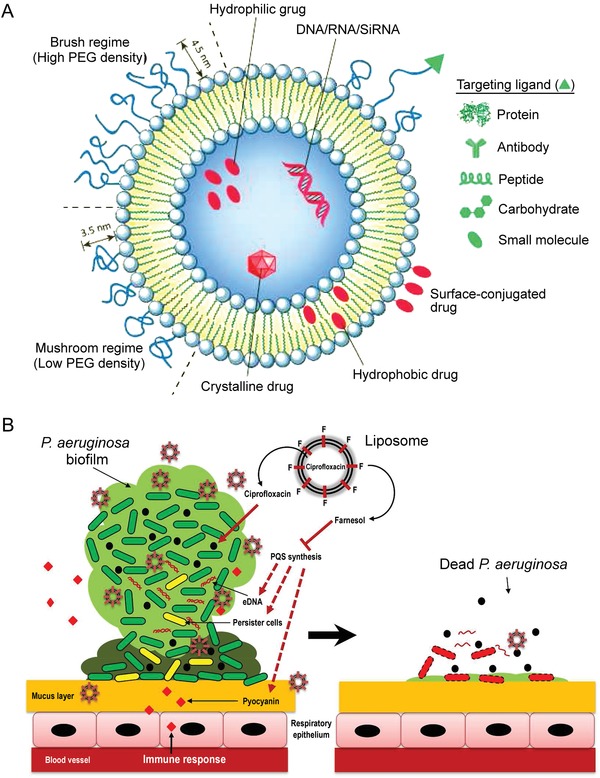
Nanodelivery strategies. A) General structure of a liposome. Reproduced with permission.^297^ Copyright 2014, Elsevier. B) Proposed killing mechanisms and summary of disruption of *P. aeruginosa* biofilms by farnesol‐ and ciprofloxacin‐loaded liposomes. Reproduced with permission.^301^ Copyright 2016, ACS Publications (further permissions related to the material excerpted should be directed to the ACS).

Recent advances in nanomedicine have been perceived as potential novel approaches for the treatment of bacterial infections.^295^ This may ultimately expand the life span of contemporary antibiotics. Phagocytes usually recognize and eliminate bacteria. However, some bacteria can survive within phagocytes, with the latter providing a haven for evading the antibacterial activity of antibiotics.^296^ Under such circumstances, nanocarriers containing antibiotics have the potential to be delivered into the infected phagocytes to eliminate the surviving intracellular bacteria. Different nanoformulations, such as liposomes and polymeric nanoparticles, have been developed for this purpose. They have been administered as drug delivery carriers and reported to improve the therapeutic index of the encapsulated agents while reducing drug toxicity (**Figure** **5**A).^297^ Liposomes are spherical lipid vesicles ranging from nanometer to micrometer in size. Their targeted delivery to host cells, typically as vehicles for targeting drug molecules into macrophages, have demonstrated potential in the treatment of microbial infections.^298^ Liposomes composed of oleic acid exhibit potent bactericidal activity against MDR *P. aeruginosa*.^299^ Their therapeutic effect is further enhanced with polyethylene glycol modification.^300^ Codelivery of farnesol, a fungal QS molecule, and ciprofloxacin via a liposomal formulation results in disrupting antibiotic‐resistant *P. aeruginosa* biofilms by enhancing biofilm killing at significantly lower antibiotic doses (Figure 5B).^301^ In addition, liposome formulations exhibit enhanced therapeutic effects against MRSA.^302^ The manner in which polymer‐augmented liposomes is designed is critical for antibiotic delivery for the treatment of intracellular macrophage infections.^303^ Improved encapsulation is achieved with the use of chitosan for coating liposomes for the intravenous delivery of vancomycin hydrochloride. The overall therapeutic efficacy is augmented while side effects such as nephrotoxicity are reduced.^304^

The loading efficacy of liposomes should be taken into consideration when choosing different types of liposomes. For example, chloramphenicol‐loaded,^305^ azithromycin‐loaded,^306^ and oxacillin‐loaded^307^ liposomes demonstrate different antibacterial activity and biocompatibility when used for treatment of MRSA‐infected dermatologic conditions. Liposomes incorporating sodium deoxycholate, a bile salt with penetration enhancing capacity, have been used to enhance the oral bioavailability of loaded drugs.^308^ Positively charged cationic liposomes are highly adept at biofilm targeting because of their interaction with negatively charged biofilm surfaces.^309^ Deformable liposomes have been used experimentally for transdermal delivery of piroxicam, a nonsteroidal anti‐inflammatory drug, for topical treatment of inflammatory skin conditions.^310^ More recently, drugs‐in‐micelles‐in‐liposomes have been developed to prevent drug leakage from the soft lipid layer of deformable liposomes, which can retain a loaded drug for up to two months.^311^

Despite these advantages, there are some drawbacks associated with the development of antibiotic‐loaded liposomes, such as instability of the vesicles and low drug encapsulation efficacy. Polymeric nanoparticles have been developed as alternative nanoformulation platforms to improve stability and drug loading. Polymeric particles reported in the literature are typically derived from poly(lactic‐*co*‐glycolic) acid (PLGA) or chitosan to form the so‐called lipid–polymer hybrid nanoparticles.^312^ Similar to liposomes, these nanoparticles are biocompatible and biodegradable but are more chemically and physically stable.

Poly(lactic‐*co*‐glycolic) acid is a copolymer of lactic and glycolic acid and is approved by the FDA for use in various drug delivery systems. In particular, PLGA nanoparticles appear to be promising for delivery of antimicrobials against lung infection caused by *P. aeruginosa*.^313^ The use of PLGA nanoparticle formulations also circumvents the limitation of amikacin, an effective anti‐*Pseudomonas* antibiotic but with high toxicity. The nanoparticles exhibit no toxicity against macrophages^314^ and possess both antibacterial and anti‐biofilm activities.^315^ Administration of PLGA nanoparticles may be helpful in overcoming treatment bottlenecks, such as infiltrating the tight mesh of biofilm/mucus in cystic fibrosis lungs with *P. aeruginosa* infection,^316^ and penetrating pathogen‐resident macrophages to treat chronic intracellular *K. pneumoniae* infection.^317^

Chitosan is a cationic, nontoxic, linear polysaccharide biopolymer that is produced via deacetylation of chitin. Chitosan has been used as an antibacterial vehicle in many studies. Chitosan nanoparticles, prepared by using tripolyphosphate as drug delivery carriers,^318^ offer many advantages and exhibit higher antibacterial activity especially against MDR Gram‐positive bacteria.^319^ Chitosan nanoparticle s have the capability to penetrate the mucus in *P. aeruginosa* related infections^320^ and possess negligible toxic side effects.^321^

Nanotechnology research has come a long way in the past decade. Lots of efforts have been made to maximize the advantages and resolve the limitations associated with existing nanoparticle technology.^322^ Because of their excellent biocompatibility and efficient surface functionalization capacity, silica nanoparticles have been used to prepare nanoparticle‐stabilized capsules for eradication of pathogenic *P. aeruginosa* and MRSA strains of clinical isolates in established bacterial biofilms.^323^ The overall progress in antibiotic delivery highlights the need to identify new systems with enhance properties, such as the use of lipid–polymer hybrid nanoparticles^324^ and lipid–dendrimer hybrid nanoparticles.^325^ These results illustrate the potential to utilize novel nanoparticles for loading of antibiotics.^326^

### 6.2 Nanoformulations for Antimicrobial Treatment

Silver has a long‐standing track record in medicine, with well‐documented antimicrobial activities and well‐understood molecular mechanisms of action. These mechanisms include disruption of cell wall or membrane, interruption of energy transduction, inhibition of enzyme activity, inhibition of DNA synthesis, production of reactive oxygen species, and acting against biofilms.^327^ Collectively, there is great potential for silver nanoparticles (AgNPs) to be used as antimicrobials to eradicate pathogenic bacteria^328^ and drug‐resistant infections caused by biofilms.^329^ Silver‐containing nanoparticles such as carboxymethyl tamarind polysaccharide‐capped AgNPs,^330^ chitosan‐capped AgNPs,^331^ gum Arabic‐capped AgNPs,^332^ polyvinylpyrrolidone‐capped AgNPs,^333^ and titanium dioxide‐capped AgNPs^334^ show inhibitory activities against ESKAPE bacteria and reduced toxicity to mammalian cells. Novel methods of synthesizing metal nanoparticles have attracted much attention. For example, biosynthesis of AgNPs using silver‐tolerant *Bacillus cereus* is an energy conservative green synthesis method that produces fewer toxic by‐products.^335^ Recent studies demonstrate strong synergistic effects between AgNPs and various antibiotics.^336^ Despite immense interest in this area, more in‐depth testing using rigorous production control, standardized toxicity assay and complementary intracellular processes are required before these nanoparticle‐based antimicrobial agents may be considered safe for clinical use.

2D nanomaterials have attracted increasing attention as antibacterial agents because of their multiple interaction mechanisms with bacterial membranes. For example, molybdenum disulfide comprises a monolayer of transition metal atoms (Mo) sandwiched between two parallel chalcogenide atomic (S) layers. The antibacterial property of molybdenum disulfide is attributed to the generation of oxidative stress which causes rapid depolarization of bacteria cell membranes.^337^ Molecular dynamics simulation revealed that molybdenum disulfide nanosheets disrupt the integrity of bacterial lipid membranes by creating dents on their surface and extracting phospholipids to undermine membrane integrity.^338^ Graphene is a sheet of single‐layer sp^2^ carbon that is tightly packed into a 2D crystal.^339^ Graphene and graphene oxide have evolved as a new generation of antibacterial agents. The bactericidal activity of graphene oxide is predominantly attributed to its unique chemical and physical properties, its extremely sharp edges and the generation of reactive oxygen species that damage bacteria cell membranes.^340^ Graphene oxide shows in vitro antimicrobial and anti‐biofilm efficacy against *S. aureus* and *P. aeruginosa*,^341^ and effectively control of MDR *K. pneumoniae* in both macrophages and animal infection models.^342^

Nanoparticles with bimodal antibacterial activities have frequently been reported in the literature. The term bimodal may be interpreted as the antibacterial action of hybrid metal nanoparticles with contact‐killing and release of metal ions, such as the use of AgNPs and ZnO.^343^ Incorporation of nanomaterials, such as Ag, is a functionalization method for introducing synergistic antimicrobial effects. Reduced graphene oxide coated with silver nanoparticle shows better antimicrobial activity against *S. aureus* at a lower concentration compared to those without the use of AgNPs.^344^ Nanocomposites comprising PLGA‐chitosan mats functionalized with graphene oxide‐Ag are capable of effectively inactivating *P. aeruginosa* and *S. aureus*.^345^ Bimodal antibacterial activities may be achieved by combining antibiotics elution with contact‐killing, such as in the case of quaternary ammonium silane‐grafted hollow mesoporous silica nanoparticles that are loaded with metronidazole for eliminating *S. aureus*.^346^ Nevertheless, the data are mainly from preclinical research and future work is required to bring the nanodelivery system more closely to its clinical realization. In contrast to the beneficial outcomes, negative effects of nanoparticles raise some safety concerns. Reports suggest that nanoparticles can pass through the blood–brain barrier, cause alteration of autonomic cardiac control, and lead to DNA damage. Various physicochemical factors may cause cytotoxicity, such as chemical nature, degradability, surface properties, surface charge, particle size, and shape.^347^, ^348^

## 7 Bacteriophage Therapy

**Figure 6:**
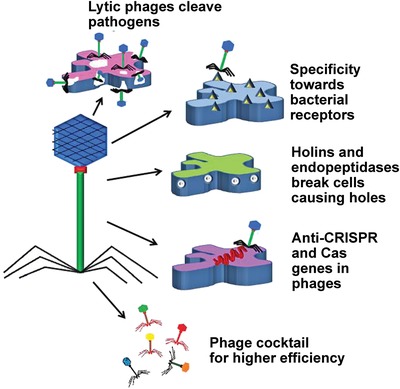
Different strategies of using bacteriophages to combat pathogens. CRISPR: clustered regularly interspaced short palindromic repeats; Cas genes: CRISPR‐associated genes. Reproduced with permission.^351^ Copyright 2016, Springer Nature.

Not only are antibiotics becoming increasingly ineffective because of bacteria resistance but also their overuse may cause dysbiosis in the gut or result in secondary infections. Bacteriophages (phages) are viruses that kill bacteria. Lytic phages are a) specific to their hosts, b) constantly coevolving with new pathogenic bacterial variants, and c) self‐regulating, self‐limiting, and self‐dosing at the site of infection (**Figure** **6**).^349^, ^350^, ^351^ Several strategies and applications of phage therapy have been reported to combat MDR pathogens,^351^ especially *P. aeruginosa*, *E. coli*, and *S. aureus* in clinical trials.^352^

A number of Phase I and II phage therapy safety trials have been concluded, showing no notable safety concerns associated with the use of phages.^353^ These favorable trial endpoints pathed the execution of Phase III clinical trials. Phages may be used to combat infectious diseases via a) phage‐derived proteins as antibacterial agents and b) phages as biosensors in pathogen detection.^354^ More research is required to develop rubrics for evaluation of the safety requirements associated with realistic application formulations,^355^ promote phage delivery (stabilization and encapsulation),^356^ and target disease selection.^357^ A recent study found that phages can attach to bacteria surface receptors that are required for pathogenesis and result in attenuated and phage‐resistant mutants which fail to produce disease.^358^ Phages have been reported to be efficient in the eradication of biofilms.^359^ “Phage cocktails” (mixtures of multiple phages that target the same host) have been developed to combat phage resistance. Commercial phage cocktails have demonstrated usefulness in the killing of various clinical isolates of *P. aeruginosa* and *S. aureus*.^360^ The combination of phage therapy and antibiotics can reduce the evolution of bacteria resistance for several reasons.^361^ Although bacteria may develop resistance to antibiotics and phages simultaneously, these strains are considerably less pathogenic. Moreover, double‐resistant mutant strains are rare because of trade‐offs between resistance mechanisms. A cocktail of natural lytic bacteriophages PP1131 (12 lytic anti‐*P. aeruginosa* bacteriophages) is active against *P. aeruginosa*‐induced experimental endocarditis and is highly synergistic with ciprofloxacin.^362^ Application of phage OMKO1 to treat chronic *P. aeruginosa*‐induced infection of an aortic Dacron graft produces no evidence of recurrent infection, which indicates a synergistic effect between phage and antibiotic therapies.^363^ A similar synergistic effect is observed when phage KARL‐1 and conventional antibiotic (meropenem, ciprofloxacin, or colistin) are administered together against MDR *A. baumannii*.^364^ Although a number of studies support the combination approach with in vitro experimental evidence, negative interference or neutral effects have also been reported.^365^ The choice of phage type and antibiotic as well as their mixing ratios require further fine tuning.

Despite the apparent advantages of phage therapy, there are potential untoward side effects identified from in vivo research.^366^ Both innate and adaptive immunity are involved in the clearance of phages from the body.^367^ Hence, there is a conceivable concern on rapid clearance of phages via immune recognition. In addition, phages can spread virulence factors among bacterial populations and may develop host resistance.^368^ Hence, drug monitoring is of great significance for clinical application. Detailed investigations on the dosage of phages and individualized phage preparation are also required.^369^ In a mouse model with MDR *P. aeruginosa*, neutrophils act synergistically with both phage‐sensitive and emergent phage‐resistant variants to clear infection, a phenomenon that has been coined “immunophage synergy.”^370^ Although this synergistic effect challenges conventional thinking that success of phage therapy is attributed to bacterial submissiveness to phage killing, more work is needed to understand all the mechanisms involved. Resistance to a phage may also occur if the bacteria surface receptor mutates or is lost, Hence, treatment of MDR infections with phage cocktails has been touted as a solution that only delays but does not eliminate the emergence of phage‐resistant mutants.^352^

Phage therapy may be conceived as a commercial cocktail or a “personalized” treatment option to an individual patient.^371^ Phage therapy based on a personalized approach is specific. Tailored phages are collected after their isolation and identification of the causative pathogens for each patient. Some investigators suggest that the available regulatory framework should also contain the patient's clinical condition and the phage administration route and dosage.^372^ For example, to reduce the bacterial burden of *K. pneumoniae* in the lungs of mice, intranasal administration is more practical than intraperitoneal administration of lytic phage 1513 for local infections.^373^

The recent upsurge in the number of clinical trials and case reports is indicative of the interest and concerns about safety and potential efficacy of phage therapy. A five‐member phage cocktail has been formulated against *A. baumannii* in a mouse infected model; the study demonstrates therapeutic efficacy of phages which are purified from environmental sources and function in a combinatorial manner.^374^ A cocktail therapy was recently formulated successfully for a patient with disseminated MDR *A. baumannii* infection when no other antibiotic regimen was effective or therapeutic option was available.^375^ Notably, this is a personalized phage cocktail treatment in which nine administered phages were identified from the patient in the laboratory. In another case report, intravenous phage monotherapy was used for combating colistin‐only‐sensitive *P. aeruginosa* septicemia in a patient with acute kidney injury.^376^ Blood bacterial culture and C‐reactive protein turned negative and fever subsided immediately after application of the phage therapy; however, the patient subsequently died from blood culture‐confirmed *K. pneumoniae* sepsis. A specialized phage therapy center in Georgia also reported the success of topical phage therapy (*S. aureus* phage‐containing eye‐drops) with nosocomial corneal abscess and interstitial keratitis, in which cultures were positive for vancomycin‐intermediate *S. aureus* and nosocomial MRSA.^377^

Phage therapy is much more than compassionate treatment (i.e., the use of new, unapproved drugs to treat seriously ill patients when no other treatments are available) and has resulted in good clinical outcomes. Nevertheless, the few formal experimental phage clinical trials produce inconclusive results on the efficacy of phage therapy, which contradict the many successful treatment outcomes observed in historical accounts and recent case reports.^378^ To better develop phage treatment and clinical applications, well‐designed randomized controlled trials are highly warranted to further define safety and efficacy. In a series of studies related to UTIs, *E. coli* and *K. pneumoniae* strains isolated from the urine of patients were tested in vitro for their susceptibility toward bacteriophages, based on which the lytic activity of commercially available phage cocktails was confirmed.^379^ A subsequent two‐phase prospective investigation was conducted to example bacteriophage adaptation and treatment with the commercially available but adapted Pyo bacteriophage. The results indicate that adaptation cycles enhance in vitro sensitivity and no bacteriophage‐associated adverse event was detected in the in vivo pilot study.^380^ Based on those preclinical endpoints, a randomized, placebo‐controlled, double‐blind clinical trial was designed to investigate potential phage treatment for UTI.^381^ Another therapeutic trial aimed at treating *P. aeruginosa*‐infected burn wounds with Good Manufacturing Practice produced phages. In this multicenter, double‐blind, randomized Phase I/II trial, PP1131 successfully reduces bacterial burden, but at a slower pace than the standard of care treatment. Nevertheless, the endpoint highlights a favorable potential for phage therapy.^382^ The clinical implementation of phage therapy faces three major challenges: 1) the scarcity of published data and randomized clinical trials restricts its application by clinical providers; 2) manufacturing challenges; and 3) obstacles in regulatory processes.^363^

It is noteworthy that the drugs based on bacteriophage enzymes, such as lysins, may exhibit more predictable results. Three putative In the eastern Europe, phages therapy has been successfully used to treat human infectious diseases. However, due to the lack of peer‐reviewed controlled clinical trials, it is difficult to accurately assess the efficacy and safety of such therapies by western standards.^351^ For example, the Eliava Institute's “preprepared cocktail” approach involves complex mixtures of unknown phages that, according to European Medicines Agency and FDA regulations, fail to be recognized as human therapies.^383^ Constant communication between drug developers and regulatory authorities on the regulatory framework is highly encouraged, which should support further tests and studies to demonstrate safety and efficacy, and to offer appropriate flexibility to speed up the availability of phage therapy.^371^, ^384^ Phage therapy is personalized medicine with customization of medicinal products tailored to an individual patient. The pharmaceutical legislation is basically designed for regulating industrially made pharmaceuticals and large‐scale distribution. Accordingly, the regulatory procedures are hardly reconcilable with the customized phage therapy. Increasing papers support that the lawmakers should agree on appropriate regulations.^385^, ^386^ Endolysins from *Myoviridae* bacteriophage family members (LysAm24, LysECD7, and LysSi3) were shown to be able to eradicate *P. aeruginosa, A. baumannii*, and *K. pneumoniae*.^387^ The bacteriophage lytic enzymes with broad bactericidal activity demonstrated their potential in the development of therapeutic agents.

## 8 Light‐Activated Antimicrobial Therapy

Ideal anti‐infective agents are supposed to be permanent or rechargeable, user‐independent, harmless to the environment, and effective in eradicating a broad spectrum of pathogens. As a light‐based sterilization technique, ultraviolet‐C light can inactivate surface pathogens by directly damaging DNA while preserving the viability of host cells. Low‐dose ultraviolet‐C combined with chlorhexidine represents a synergistic strategy to reduce bacterial burdens in canine skin and muscle samples for MRSA, MDR *K. pneumoniae*, and *E. faecium*.^388^

**Figure 7:**
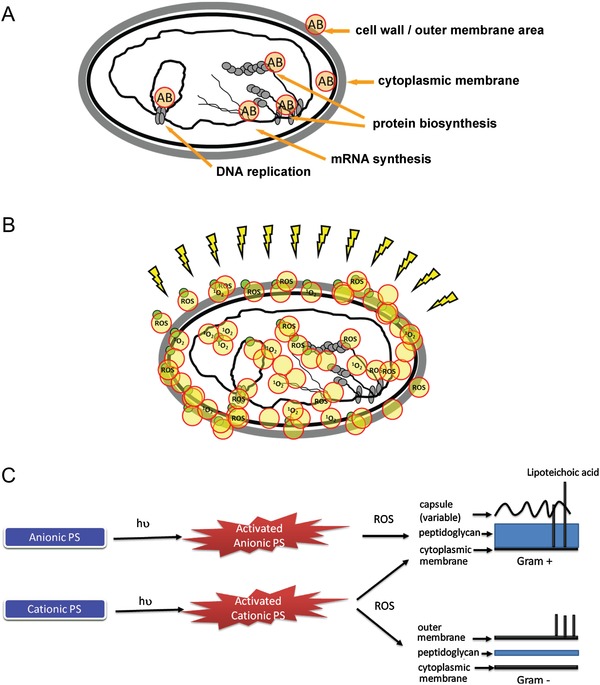
Modes of action: antibiotic versus photodynamic therapy (PDT). A) Different antibiotics (AB) react selectively with different molecules on specific organelles/structures. B) Nonspecific localization of a photosensitizer prior to illumination of the bacteria. Generation of reactive oxygen species (ROS) after light activation of the photosensitizer. Reproduced under a Creative Commons Attribution 3.0 Unported Licence.^389^ Copyright 2015, Published by The Royal Society of Chemistry (RSC) on behalf of the European Society for Photobiology, the European Photochemistry Association, and RSC. C) Schematic representation of antimicrobial PDT. Reproduced with permission.^393^ Copyright 2015, Elsevier.

Photodynamic antimicrobial chemotherapy (PACT) or photodynamic therapy (PDT) was discovered at the start of the 20th century. The technique employs harmless white light to photoinactivate both antibiotic‐sensitive and antibiotic‐resistant pathogens via the generation of cytotoxic reactive oxygen species (**Figure** **7**).^389^, ^390^, ^391^, ^392^, ^393^ The PACT technique involves three elements: oxygen, a photosensitizer, and a harmless light source. In the presence of oxygen, the activated photosensitizer transfers its energy to molecular oxygen and generates reactive oxygen species, such as singlet oxygen and hydroxyl radicals. The latter are responsible for killing of microbial cells present in their vicinity. As the key component of PACT, an ideal photosensitizer should have high photostability to minimize photobleaching, selectivity to bacterial strains, and no harmful effects on host cells.^392^ Because the outer membrane of Gram‐negative bacteria is more negatively charged, cationic photosensitizers are more effective, while anionic photosensitizers are generally only active against Gram‐positive bacteria.^393^ Photodynamic inactivation produces reactive oxygen species that can lethally damage a host of microbial biomolecules, whereas antibiotics generally just inhibit growth. Hence, the chance of resistance to PDT is considered highly unlikely.^394^ Although in vitro studies reported the killing of a wide variety of species, more emphasis should be placed on in vivo studies with carefully chosen types of infection.

For the treatment of *A. baumannii* infections, photosensitizers that have demonstrated potential include toluidine blue O,^395^ TiO_2_ nanoparticles,^396^ and ZnO nanoparticles.^397^ Recent studies report the use of fullerenes to mediate PDT; in vitro and in vivo studies tested the potential application of fullerenes with iodide for targeting *A. baumannii* infection,^398^ and fullerenes with one methylpyrrolidinium group for combating *S. aureus* infection.^399^ Future studies are likely to be focused on functionalized or conjugated second generation photosensitizers. The use of single‐walled carbon nanotube‐porphyrin conjugates for visible light‐mediated inactivation reported potent antibacterial activity against *S. aureus*.^400^ Sinoporphyrin sodium‐mediated PACT demonstrated significant bactericidal activity against MDR *S. aureus* in burn‐infected mice.^390^ Similar results were obtained using porphyrin chlorin e6 with red light in a porcine eye model.^401^ Target‐oriented photofunctional nanoparticles have been designed for selectively capturing and killing of MRSA.^402^ For example, attachment of the NorA efflux pump inhibitor INF55 to methylene blue enhances antimicrobial photodynamic inactivation of MRSA in vitro and in vivo.^403^ Cationic porphyrins and their derivatives have been used as antimicrobial photosensitizers against MRSA and *P. aeruginosa* in vitro^404^ with dose‐dependent efficacy in vivo.^405^ Conjugation of pentalysine to a zinc phthalocyanine‐based second generation photosensitizer via the use of carbodiimde enhances the efficacy of PACT, with increased cell uptake of the photosensitizer in a murine *S. aureus* skin infection model.^406^ In similar studies, conjugation of tertiary amine to produce positively charged phthalocyanine photosensitizers results in negligible toxicity in human cells; the cationic photosensitizers are more effectively bound to bacteria compared to their neutral or anionic counterparts.^407^ Photodynamic therapy has found important use in combating clinically relevant biofilms and has been tested in several clinical trials of localized biofilm infections in humans.^408^ Criteria in the design of novel photosensitizers include agents with high water solubility, selective attachment to pathogens, and negligible toxicity to human cells.^409^ Combining PDT with clinical antimicrobials improves eradication of *S. aureus* and *E. coli*,^410^ XDR, and even PDR *A. baumannii*.^411^

Photothermal therapy refers to laser‐induced physical destruction of bacteria integrity by strong light absorbers such as gold nanoparticles or carbon nanotubes.^412^ The administration of IgG‐gold nanoparticles with laser irradiation results in extended and selective bacterial death that occurs in a dose‐dependent manner.^413^ Antibiotic‐loaded, antibody‐conjugated, polydopamine‐coated gold nanocages have been tested as a means of achieving highly targeted, laser‐assisted photothermal effects. The results demonstrate high efficacy in synergistic killing of *S. aureus* and *P. aeruginosa*, and as proof‐of‐concept for eradication of ESKAPE pathogens in established biofilms.^414^ While the appropriate antibody and antibiotic combination requires meticulous selection, the mechanism behind this highly desirable therapeutic synergy is thought to be caused by the cationic gold nanocages penetrating deeply into biofilms and the generation of laser‐induced vapor nanobubbles. The latter are hypothesized to function by subtly but significantly expanding the spaces between sessile cells, thereby enabling better penetration of antibacterial agents.^415^ However, there is inadequate evidence on the toxicity of such an antibacterial system on host cells in animal models or human experiments.

## 9 Other Antibacterial Agents

Bacterial resistance to available antibiotics continues to emerge in clinical practice and very few new molecules are active against MDR gram‐negative pathogens.^65^ Antibacterial agents with attractive properties, including additional binding to target sites and novel modes of action, become the primary targets of many investigations.

Several small molecules that enhance the effects of antibiotics as adjuvants have been reported. Different mechanisms are purportedly involved, including inhibition of antibiotic modification, target modification, signaling pathways, biofilm formation, and enhancement of antibiotic uptake.^416^ For example, small molecule interference via bacterial communication and signaling pathways, including quorum sensing and two‐component signal transduction systems, has been discussed comprehensively.^417^, ^418^ 2‐Aminoimidazoles are small molecules that possess adjuvant activity to inhibit and disperse bacterial biofilms. Aminoimidazole/triazole conjugate was reported to resensitize conventional antibiotics with MRSA and multidrug‐resistant *A. baumannii*.^419^ Melander's labs have been working on the use of bis‐2‐aminoimidazole adjuvants in the case of cystic fibrosis patients with *P. aeruginosa* infection. This new method exhibits reduction in minimum inhibitory concentrations of azithromycin as high as 1024‐fold in vitro and displays activity in vivo in a Galleria mellonella infection model.^420^ They also identified some derivatives of tryptamine, which are capable of disarming colistin resistance in *A. baumannii, K. pneumoniae*, and *E. coli*.^421^ Blackledge's group described that amoxapine,^422^, ^423^ an FDA‐approved tricyclic antidepressant, and loratadine,^424^ an FDA‐approved antihistamine, could potentiate β‐lactam antibiotics in MRSA with different mechanisms of action. Important structural features of these compounds require further exploration.

Plants used in phyto‐medicinal practices against infections represent a promising source of bioactive compounds. Many studies have demonstrated the potential of natural products and their derivatives in possessing microbicidal action, not only on planktonic bacteria but also in inhibiting QS activity^425^ and biofilm formation.^426^ Antimicrobial properties in plants are attributed to the presence of active compounds and peptides present in their defense systems which are similar to human AMPs in structure and function.^427^ Plant extracts have the ability to bind to protein domains and affect key events in the pathogenic process, Thus, they can act as either antimicrobial agents or resistance modifiers.^428^ A number of plant active compounds have been identified that may become useful therapeutic tools with antibacterial activity against both Gram‐positive and Gram‐negative bacteria.^429^ However, challenges exist in that the unusual molecular architectures of these phytochemicals may require new synthetic strategies and technologies.^430^

Essential oils are volatile, natural fragrant liquids that are extracted from different parts of plants, especially leaves and flowers. The activity of essential oils is commonly ascribed to the perturbation of cell membrane structural integrity, leading to bacterial cell death. Because of the advantage of combining essential oils and AMPs, a centralized resource, specifically for anti‐*S. aureus*, was created to facilitate comprehensive investigation of their activity associations and combinations.^431^ Continued interest in the antibacterial activity of various essential oils and the synergism between them and antibiotics may be found in the literature.^432^ Essential oils derived from *Ferula ovina* 433 and *Sideritis romana L. subsp. Purpurea* 434 show potential for use as alternative remedies for the treatment of infectious diseases caused by MRSA. Those derived from *Cladanthus arabicus, Bubonium imbricatum*,^435^ and *Thymus vulgaris* 436 exhibit antibacterial activity against *Enterobacteriaceae* isolates and also exert active effects when combined with conventional antibiotics. The synergy between essential oils derived from *E. camaldulensis* EO and conventional antibiotics is taken advantage of in the development of new treatment strategies against MDR *A. baumannii* infection.^437^ Different essential oils extracted from Mediterranean plants have been analyzed for their ability to destabilize *P. aeruginosa* biofilms at very low concentrations.^438^ Tea tree oil is a well‐known antibacterial agent which inhibits bacterial respiration and disrupts bacterial membrane permeability.^431^ This oil possesses extensive bactericidal properties against clinical strains of MRSA, carbapenem‐resistant *K. pneumoniae*, *A. baumannii*, and *P. aeruginosa*.^439^ Hence, tea tree oil represents a possible nonconventional regimen against *S. aureus* and Gram‐negative MDR bacteria.

The sensitivity of essential oils to environmental factors and their poor aqueous solubility have limited their applications in industries. Different nanoparticles have been used to encapsulate and protect the lability of these oils which could be derived from peppermint,^323^ *Carum copticum*,^440^ and *Lippia sidoides*.^441^ More recently, nanoemulsions prepared from *Cleome viscosa* essential oil show potent biocidal activities against extended‐spectrum β‐lactamase producing *E. coli*, *K. pneumoniae*, *P. aeruginosa*, and MRSA.^442^

Over the past decade, promising results have been obtained for the antibacterial activity of natural flavonoids.^443^ Published data highlight the excellent pharmacological potential of flavonoids and their ability to combat MDR bacterial infections.^444^ The focus of recent studies is on examination of antibacterial mechanisms. The aglycone forms (i.e., compound that remains after the glycosyl group on a glycoside is replaced by a hydrogen atom) of flavonoids in subminimal inhibitory concentrations inhibit biofilm formation by *S. aureus* strains that overexpress efflux protein genes.^445^ Paradoxically, flavonoids isolated from Kenyan plants were found to be substrates of bacterial efflux pumps when used against MRSA; the flavonoids were recommended to be together with efflux pump inhibitors.^446^ These findings indicate the ambiguous interactions of these bioactive compounds with bacteria. Another proposed mechanism against *P. aeruginosa* is the inhibition of fatty acid syntase‐II β‐hydroxyacyl‐(acyl‐carrier‐protein) dehydratase complex that was mediated by flavonoids extracted from *Trianthema decandra*.^447^ Because synthetic derivatization of flavonoids produces stronger antibacterial effects, the chemical process appears to have potential for the design of new antibacterial agents.^448^ Anti‐MRSA activity was investigated with potential synergy of the desired flavonoid with β‐lactam antibiotics such as oxacillin^449^ and cefazolin.^450^ The test results indicate that some flavanoids are capable of restoring the activity of antibiotics and augmenting their killing effect.

The exploration for plants for their potential therapeutic utility requires further drug research and development processes, which include molecular docking analysis, herbal selection, and bactericidal activity assessment in sequence.^451^ *Scutellaria barbata*, a Chinese herb, exhibits in vitro and in vivo activity against extensively MDR *A. baumannii*, with better antibacterial effect than the use of colistin alone.^452^ The aqueous extract of *Lawsonia inermis* L. (henna) contains antibacterial components against *S. aureus* and *K. pneumoni*a; the abundant free hydroxyls may have contributed to its antibacterial activity.^453^ Chlorogenic acid, a component of burdock root extract, possesses anti‐biofilm and anti‐β‐lactamase activities against recalcitrant *K. pneumoniae*.^454^ Extracts from *Kalanchoe fedtschenkoi*, a thick‐leaved succulent, possess growth inhibitory effects against *A. baumannii, P. aeruginosa*, and *S. aureus*.^455^ Two benzophenanthridine alkaloids, dihydrochelerythrine and N‐methylcanadine, possess anti‐MRSA activity and may serve as potential leads for the design of new bioactive compounds.^456^ Low molecular weight natural products, mainly polyphenols and terpenes, have been reported as promising adjuvants for antimicrobial drugs, although most works included only in vitro assays.^457^

Clay minerals are naturally occurring layered phyllosilicates with stable crystalline structures. They have a long history in the treatment of human diseases and appear to have excellent therapeutic properties in the laboratory. Kisameet clay has been reported to possess broad‐spectrum antibacterial activity against a panel of MDR ESKAPE strains.^458^ To continue this search, detailed in vivo investigations have to be performed, such as identification of functional molecules, administration route, optimal concentration, and cytotoxicity.^459^ Boho soil cure, containing *Streptomyces sp*. *myrophorea*, inhibits many MDR ESKAPE pathogens including carbapenem‐resistant *A. baumannii*, vancomycin‐resistant *E. faecium*, and MRSA.^460^

Human milk oligosaccharides (HMOs) serve as novel chemical scaffolds for the development of antibacterial agents. The HMOs have important nutritional and biological activities that guide the development of the immune system and the proper neonate microbiome. In this manner, HMOs help protect against pathogen colonization and reduce the risk of infection.^461^ More recently, HMO was reported to possess anti‐biofilm and antimicrobial activities against some ESKAPE pathogens.^462^ The mode of action of HMO activity may be divided into two categories: selective metabolism of HMOs by symbiotic bacteria and direct interaction with pathogens to increase cell permeability. This renders HMO an effective adjuvant for intracellular‐targeting antibiotics.^463^ Nevertheless, the limited availability of HMOs may be the greatest barrier to research.

Repurposing well‐characterized drugs with clinically safe and pharmacology profiles is an untapped source of new antimicrobial drug discovery^464^ and may contribute to reducing problems associated with antibiotic resistance.^465^ In 2015, ebselen was identified through a library screening of FDA approved drugs. The therapeutic efficacy of ebselen was evaluated in a murine model of skin infection. Eebselen demonstrated potent bactericidal activity against MDR clinical isolates of *S. aureus*, including MRSA and vancomycin‐resistant *S. aureus*.^466^ Ebselen, which also possesses anti‐inflammatory properties, may act as an antimicrobial agent through inhibition of protein synthesis and subsequent inhibition of toxin production in MRSA.^467^ These findings prompted researchers to investigate the antibacterial activity of ebselen‐inspired compounds. In 2016, ebsulfur, in which the selenium in ebselen is replaced by a sulfur atom because of the concerns on selenium toxicity, was found to possess good antibacterial activity against *S. aureus* clinical isolates.^468^ Because ebselen lacks major activity against Gram‐negative ESKAPE pathogens, some selenazolinium derivatives were investigated in 2017, which displayed significant activities against Gram‐negative pathogens.^469^ In 2018, ebselen was found to be effective as ramoplanin in reducing bacterial shedding and burden of VRE in a murine model.^470^

High‐throughput screenings have been used successfully to identify antibacterial inhibitors of clinical pathogens, such as *A. baumannii*.^471^ Screenings are used to rigorously interrogate a high volume of potential drug candidates, with the anticipation of high attrition rates. Despite such efforts, the process of converting inhibitors of purified target enzymes into compounds with whole‐cell activity is the most challenging because of a lack of understanding of the mechanisms of cell penetration.^472^ Natural scaffolds represent a very promising chemical family that may efficiently generate new drug candidates.^473^ For example, a highly modular synthetic strategy has been employed for the construction of natural and substituted tetracyclic meroterpenoids, from which strongylin A was identified to possess potent activity against MRSA.^474^

Revisiting unexploited antibiotics is a potential strategy for identification of novel antibacterial drug candidates.^475^ Kibdelomycin, identified in the 1950s, is the first class of natural‐product‐derived bacterial gyrase inhibitors which exhibit broad‐spectrum antibacterial activities.^476^ Further studies on kibdelomycin resulted in the identification of three kibdelomycin derivatives that reveal an early structure–function relationship.^477^ Kibdelomycin utilizes dual enzyme inhibition and multisite “U shape” binding mode to inhibit DNA topoisomerase II (gyrase B and topoisomerase IV), which accounts for its lack of cross‐resistance and low frequency of resistance.^478^ Extended profiling of kibdelomycin shows bactericidal activities against ESKAPE pathogens such as *A. baumannii* and *P. aeruginosa*.^479^ Reevaluation of known antibiotics facilitates the design of analogs with better properties.^480^

## 10 Status Quo of Antimicrobial Agents

**Figure 8:**
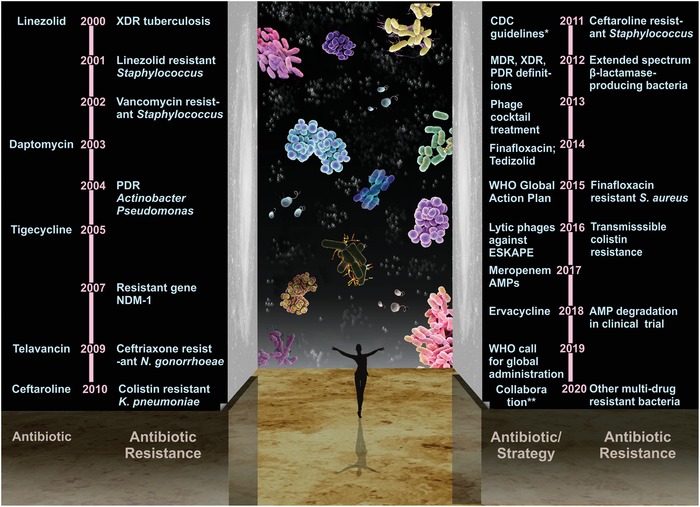
Antibiotics and strategies against multidrug‐resistant bacteria in the past 20 years. Humans are belittled and vulnerable in their confrontation with the expanding galaxies of multidrug‐resistant bacteria. Introduction of a new antibacterial agent was soon followed by a corresponding sophisticated bacteria resistant strategy which renders the drug nonfunctional against the new resistant strains. AMP: antimicrobial peptide; CDC: Center for Disease Control & Prevention; MDR: multidrug‐resistant; *N. gonorrhoeae*: *Neisseria gonorrhoeae*; NDM‐1: New Delhi metallo‐beta‐lactamase 1; PDR: pan drug‐resistant; WHO: World Health Organization; XDR: extensively drug‐resistant. *CDC guidelines for the prevention of perinatal Group B *Stretococcal* diseases; **Collaboration between pharmaceutical companies and national/international policy‐making bodies.

The battle between humans and disease‐causing bacteria has never stopped since the emergence of human civilization 3 million years ago. Prior to the 1940s, drugs that can effectively treat bacterial infections with little side effects were not yet available. Handwashing was perceived as the most effective means for preventing infections in that dark era. After the introduction of penicillin as a therapeutic agent against staphylococcal infection, the U.S. Health Director William Stewart boldly declared that the day for complete eradication of infectious diseases was not far off. However, it was not long after his ostentatious announcement that scientists discovered that up to 90% of the staphylococcus species developed resistance to penicillin. Thenceforth, every time a brand‐new antibiotic was introduced, bacteria would come up with a corresponding sophisticated resistant strategy (**Figure** **8**). The result of this abiding war is the emergence of ESKAPE pathogens armed with multiple resistance mechanisms that are responsible for the majority of recalcitrant bacterial outbreaks in nosocomial settings.^481^

Even though the development of antimicrobial agents against ESKAPE is rapid, the resistance mechanisms against the antimicrobial agents are emerging at the same time, causing pandemic and enormous clinical and financial burdens on global health care systems. Decisive actions that require significant commitment and enforcement have never been popular even if lives can be saved. Sadly enough, the World Health Organization published a report in 2017 proclaiming that the world's antibiotic pipeline was drying up.^64^ Although new antibiotics such as oxazolidinone may be promising in combating ESKAPE pathogens (Section 3), they share the same property as their predecessors that have been eliminated from the list, in that they rely on a single antibacterial mechanism. The timeline for these new antibiotics to become ineffective in eliminating the ever evolving ESKAPE pathogens is foreseeable. Humans are belittled and unconsciously becoming more passive and vulnerable in their confrontation with these unfathomable galaxies of drug‐resistant bacteria (Figure 8). For the antiresistance and antivirulence drugs described in Sections 4 and 4.3, respectively, they only abolish the protective mechanism of the bacteria rather than killing the bacteria per se. This explains why these drugs/inhibitors are only classified as adjuvants of antibiotics in clinical practice.

There are several bottlenecks that challenge their clinical translation: cost, the community's anxiety associated with opening a potential Panadox box and the mode of delivery in commercial applications. We will interpret them one by one in the following text.

In terms of cost, production of antimicrobial nanoparticles at an industrial scale is usually conducted by solid‐phase particle size reduction, liquid‐phase synthesis, or gas‐phase synthesis. Apart from economic considerations, each process suffers from limitations ranging from poor property control to the introduction of contaminants into the product.^482^ To circumvent these issues, a humongous amount of research dollars has been invested in designing better nanoparticles or better delivery approaches, as well as streamlining the scalability of commercialization processes. There are two approaches to address the cost issue.^483^ One proposed approach is to use low‐purity nanomaterials without significantly compromising efficiency. This is because much of the production cost is related to separation and purification. Low‐cost nanomaterials should be explored for potential applications in antimicrobial therapeutics. Alternatively, cost effectiveness may be improved by retaining and reusing nanomaterials. For AMPs, various approaches are already offered, including insertion of non‐natural or d‐amino acids, introduction of peptide‐mimetics, peptide cyclization, acetylation, and amidation at the N‐terminus to avoid peptide degradation or the design of short peptides to reduce production costs.^190^

Apart from the cost involved in product development, the rose garden promise of novel AMPs or other novel antibacterial materials has been overshadowed at large by continuing questions on the spread of bacteria resistance among communities and the environmental pollution. The most notable characteristic of these pathogens is their strong ability to mutate. This is because bacteria reproduce so quickly, producing the next generation within minutes. Adaptational changes by plasmids that occur in one area can quickly spread to other areas. Indiscriminate prescription of antibiotics over the past few decades has led to the post‐antibiotic era, where no antibiotics are capable of fighting diseases. One cannot guarantee, based on accumulating evidence, that such an episode will not be recapitulated with the clinical application of AMPs, HMOs, or other antibacterial agents. This has led to the community's anxiety in opening another Pandora box. For example, bacteria avoid AMPs by changing the structure of the binding site for AMP on the bacterial membrane or producing proteases to eliminate the AMPs. Although metal nanomaterials appear to directly destroy the protective membrane of bacteria and are difficult to be digested by these microbes, long‐term metal exposure to the environment will cause air or soil pollution. Accordingly, hybrid approaches should be encouraged in future drug design. For example, AMPs and metal nanomaterials may be used in combination to penetrate the membrane and destroy the genetic structures such as plasmids simultaneously. This strategy can make full use of the characteristics of nanomaterials such as nanocarriers or transporters to increase the stability of AMPs, reduce the possibility of plasmid diffusion, and prevent mutation. In addition, combined drug design strategies such as the combination of function design and material recycling should be considered. For nanoparticles that release toxic metals such as nanostructured Ag and metallic quantum dots, it is important to control their dissolution by using stabilizing coatings or optimizing nanoparticle shape and size. Depending on the application scenario, barrier technologies such as filtering membranes and magnetic separation may be required to recover nanoparticles and prevent their release into the environment.

Common limitations of antibacterial therapy include targeted delivery inside the human body, toxicity, and pollution to the environment. These issues have become huge hurdles in combating bacteria in clinical translation. A thorough understanding of the structure of antimicrobial agents and their interaction with bacteria as well as host cells is urgently needed. Currently, two evolving technologies are involved in the selection of candidate drug in pharmaceutical industry: network pharmacology and functional genomics profiling. Utilizing these platforms will enable more informed decisions to be made on candidate drug selection and can maximize or predict therapeutic potential prior to clinical testing.^484^ Recently, decoy fluorophores that bind to bacterial membranes but do not transport target compounds have been developed for studying membrane transport by ESKAPE pathogens. They have been incorporated in screening assays to identify potential antimicrobial agents that inhibit bacteria membrane transport.^485^

The aforementioned uncertainties extend to how government agencies regulate the creation, production, and distribution of antimicrobial agents. Major international and national initiatives aimed at financially incentivizing the research and development of novel antibiotics have been implemented.^486^ Although these promising agents are appealing for off‐label use, researchers and clinical practices should be cautious because high‐quality clinical data for off‐label regimes are limited.^487^

In 2013, European Medicines Agency set an invitation‐based workshop to discuss aspects of the development of antibacterial medicinal products, including those targeting MDR pathogens and those with narrow action spectra.^488^ Several important recommendations were delivered, including a) an option based upon noninferiority is essential because showing superiority on a hard end‐point is not routinely possible and b) both broad and narrow spectrum agents need at least one standard pivotal noninferiority study in at least one infection type.^488^ In 2016–2017, a series of meeting was held of European Medicines Agency, the Japanese Pharmaceuticals and Medical Devices Agency, and FDA. A consensus was reached by the three agencies to explore common regulatory approaches and align their data requirements and clinical trial design.^489^

Unnecessary use of broad‐spectrum antimicrobials can result in delayed healing, which is the strongest predictor of mortality with ESKAPE pathogens.^490^ The development of narrow‐spectrum antibiotics that do not generate cross‐resistance in nontargeted pathogens, and elicit abrogated or reduced collateral damage upon the host microbiome, is therefore an attractive approach.^491^ Clinicians need to accurately diagnose infections to plan patient treatment and improve antibiotic stewardship. Researchers are working on novel technologies to rapidly extract lipids from bacterial membranes for rapid identification of ESKAPE pathogens.^492^

## 11 Perspectives and Future Directions

For the past couple of decades, ESKAPE pathogens are responsible for the lion's share of nosocomial infections, acquiring resistance and virulence determinants that have enabled them to affect seriously how medicine is practiced in the modern hospital setting. Notwithstanding all good intentions by policy makers to control antibiotic usage,^493^ there is little doubt that current strategies in combating antibiotic resistance is grim. This review comprehensive summarized the recent alternative antibacterial agents such as AMPs, HMOs, metal nanoparticles, antibacterial polymers, or even probiotics, demonstrating good performance against ESKAPE pathogens in vitro, and their application to date is predominantly at an academic research level and at best at the preclinical setting.^487^, ^494^ We believe that our article will play a guiding role in the update or creation of future strategies for combating ESKAPE.

After summarizing so many basic researches on antibacterial, guiding them to the clinic is the key to benefiting patients. In the last five years, fewer than five Phase IV trials have been successfully completed (Table 2). Linezolid showed favorable microbiological and clinical outcomes in combating nosocomial pneumonia caused by MRSA in patients older than the age of 18.^495^ For younger patients, once‐daily daptomycin is relatively safe and efficacious in managing complicated skin and skin structure infections caused by Gram‐positive bacteria, including community‐acquired MRSA.^406^, ^497^ However, robust evidence is lacking for drugs targeting the other ESKAPE pathogens, especially *P. aeruginosa*, Klebsiella, and Acinetobacter. Developing narrow‐spectrum drugs targeting these ESKAPE pathogens is the most pressing and yet the most challenging task in the global war against antimicrobial resistance.

Innovative trial design and analytical methods are crucial for clinical development of drugs targeting ESKAPE pathogens, which are summarized in the following eight tips: 1) dosing regimens based on pharmacokinetic/pharmacodynamic analyses in preclinical studies as well as Phase I and II trials; 2) reassessment of old antibiotics; 3) validated external controls; 4) more sensitive end points in time‐dependent or rank‐based manners; 5) hierarchical nested‐trial designs; 6) incorporation of concomitant or historical trial data via platform trials and/or Bayesian methods; 7) very small clinical studies; and 8) rigorous quality improvement. These elements have been discussed in details in the latest white papers.^498^, ^499^ Recent evidence is promising with respect to the incorporation of these innovative elements in the clinical development of antibiotics targeting ESKAPE pathogens. For example, dosing regimens of meropenem based on pharmacokinetic and pharmacodynamic models improve clinical response in the treatment of lower respiratory tract infections caused by *K. pneumoniae* and *P. aeruginosa* in elderly patients.^500^

Bacteria are invincible. They owned life forms for billions of years, prior to the emergence of humans. In this protracted war against ESKAPE pathogens, humans should assume the humble role of the defender and design hybrid strategies by combining materials design, nanotechnology, immunity research, and other disciplines, aiming at keeping evil bacteria under their control. Only by actively combining human self‐recognition and scientific innovation can we prevent another antibacterial apocalypse.

## Conflict of Interest

The authors declare no conflict of interest.
